# Techno-economic optimization of a grid-connected solar–wind - pumped hydro hybrid system for energy and desalination in Ras Ghareb, Egypt

**DOI:** 10.1038/s41598-026-49904-2

**Published:** 2026-05-06

**Authors:** Hilmy Awad, Nadia Shukri Mohamed Abu El-Nasr, Hassan Mahmoud, Samia Abdalfatah

**Affiliations:** 1 Electrical Technology Department, Faculty of Technology and Education, Helwan University, Cairo, Egypt; 2 Department of Electrical Technology, Faculty of Technology and Education, Helwan University, Cairo, Egypt; 3 Egyptian Electricity Holding Company (EEHC), Cairo, Egypt; 4 Department of Power and Electrical Machines Engineering, Faculty of Engineering, Nahda University, Beni Suef, Egypt

**Keywords:** Techno-economic, Grid-connected, Wind turbine, Pumped-hydro storage, Desalination, HOMER, Emission, Energy science and technology, Engineering, Environmental sciences

## Abstract

The growing demand for electricity and fresh water in remote and coastal regions necessitates sustainable solutions that reduce reliance on fossil fuels. Hybrid renewable energy systems, which combine solar, wind, and storage technologies, have proven effective in ensuring a reliable supply and environmental sustainability. However, few studies have addressed large-scale hybrid applications that simultaneously meet electricity and desalination needs in coastal areas, and the integration of pumped-hydro storage with PV and wind in Egypt’s high-potential regions is underexplored. In particular, previous work has rarely incorporated realistic mixed residential, agricultural, and desalination load profiles or applied Diversity Factors to represent actual consumption behavior. This paper investigates the techno-economic feasibility of a hybrid system integrating photovoltaic (157.6 MW), wind (166.8 MW), and pumped-hydro storage (223,661 kWh) to supply Ras Ghareb, Egypt, using HOMER Pro simulations and real data for 5000 residential homes, agricultural machinery for irrigating 2000 acres of farmland, and the power demands of a desalination plant. The optimized system achieves a Renewable Fraction of 93.8% with no unmet load. Economically, the proposed system demonstrates strong performance, with a Net Present Cost of − $94.7 million, an Internal Rate of Return of 53%, and a simple payback period of 1.9 years, driven by selling surplus power to the grid. The qualitative sensitivity trends indicate that the system remains robust under reasonable variations in resource conditions and pricing assumptions. Environmentally, the system reduces annual CO₂ emissions by 291.7 million kg, SO₂ emissions by 1.26 million kg, and NO_x_ emissions by 0.62 million kg. These results also provide relevant insights for ongoing national energy and water strategies, particularly regarding renewable expansion and long-duration storage in coastal regions. The findings confirm the system’s technical reliability, financial feasibility, and environmental benefits, positioning the system as a scalable model for sustainable energy in remote and coastal regions.

## Introduction

Remote and coastal regions continue to face persistent challenges in securing reliable electricity and freshwater, largely due to their isolation from centralized utility networks. Brandoni and Bošnjaković^1^ showed that water-stressed communities require integrated approaches that simultaneously consider energy supply and water demand. In parallel, Shemer and Semiat^2^ demonstrated that Reverse Osmosis (RO) desalination, while essential for freshwater production, remains a highly energy-intensive process that necessitates stable power sources.

Energy system design in remote areas is further complicated by erratic consumption patterns. Lau et al.^3^ found that variations in fuel prices, load sizes, and financing conditions strongly influence the feasibility of renewable systems on isolated islands. To address this, Rezk et al.^4^ employed multi-criteria decision-making methods to optimize hybrid renewable configurations for higher performance. At the national level, Abubakr et al.^5^ provided an in-depth review of Egypt’s renewable energy progress, grid codes, and long-term plans, confirming the country’s strong potential for large-scale clean energy deployment.

With expanding agricultural and industrial activity, Egypt’s energy needs continue to rise. Aref et al.^6^ highlighted that optimizing the placement of high-penetration renewable systems is essential for maintaining grid stability under increasing demand. Similarly, Ma and Javed^7^ discussed how hybrid PV-wind-battery systems can maintain reliability in remote communities despite resource intermittency. Datta et al.^8^ showed that machine-learning-driven photovoltaic modeling is improving the accuracy and efficiency of PV forecasting and operation.

Energy markets also influence renewable adoption patterns. Tambari et al.^9^ showed that fluctuations in global oil prices have uneven effects on renewable development across African economies, reinforcing the need for diversified energy structures. In Egypt, Hamdi et al.^10^ applied artificial neural networks to optimize a dispatchable hybrid plant in Ras Ghareb, confirming the region’s favorable wind and solar conditions. Earlier studies by Ahmed^11^ documented the performance of the first wind farm in Ras Ghareb, while his later work^12^ provided a detailed techno-economic assessment of wind potential along Egypt’s Mediterranean coastline.

The use of hybrid renewable systems in off-grid and grid-connected applications has been widely validated. Afif et al.^13^ demonstrated that hybrid PV-wind systems can significantly reduce operating costs when appropriately sized using HOMER. Ayadi et al.^14^ further confirmed that renewable integration enhances grid resilience and flexibility in smart grid environments.

While the literature provides strong evidence for hybrid systems, a major research gap remains in the large-scale integration of solar, wind, and pumped-hydro storage for combined residential, agricultural, and desalination loads, particularly in coastal regions with exceptional renewable resources.

Although several studies have examined hybrid renewable systems in Egypt, most have focused on either PV- or wind-dominant configuration without incorporating large-scale storage. Existing work on Ras Ghareb has not evaluated mixed residential–agricultural–desalination loads using realistic demand profiles or diversity modeling. In addition, previous studies rarely compare hybrid systems against a grid-only baseline, leaving limited evidence on the relative economic and environmental value of such configurations.

This study addresses these gaps by developing and optimizing a grid-connected hybrid system for Ras Ghareb, Egypt, using detailed load modeling for 5000 households, 2000 acres of irrigation, and an RO desalination plant with a capacity of 80,000 m^3^/day. A Diversity Factor was applied to capture realistic consumption behavior and improve system precision. By incorporating Pumped-Storage Hydropower (PSHP) as a large-scale storage solution, this research evaluates a mixed-load environment that has not been previously addressed in the region, providing a direct techno-economic comparison with a grid-only baseline.

### Article contribution

This article contributes to ongoing research on hybrid renewable systems for coastal regions by incorporating realistic mixed-sector electricity demands and evaluating a PV–wind–pumped-hydro configuration tailored to Ras Ghareb. The study also provides a direct comparison with a grid-only case to clarify the relative technical and economic effects of the proposed system.

The primary contributions are:

- Use of combined residential, agricultural, and desalination load profiles, offering a more representative demand model than single-sector or synthetic profiles commonly used in earlier studies.
- Application of a Diversity Factor to mixed loads, improving the accuracy of demand estimation for multi-consumer environments.
- Techno-economic comparison between the hybrid system and a grid-only baseline, enabling a clearer understanding of cost, performance, and environmental differences.

The remainder of the paper is organized as follows: Section “Literature review” outlines Egypt’s electricity demand and describes the Ras Ghareb site; Section “Site description and resource potential” reviews national solar and wind resources; Section “Study site and research rationale” presents the study rationale and key parameters; Section “Study methodology” details the HOMER-based modeling methodology; Sections “Analysis of electrical loads” and “Hybrid system resources” analyze the electrical loads and renewable resources; Sections “Hybrid system schematic” and “Modeling and simulation of the hybrid solar / wind system components” describe the hybrid system architecture and component simulations; Section “Results analysis and ecnomic discussions” discusses the technical, economic, and environmental results; Section “Grid emission factors” evaluates emission impacts; Section “Validation and benchmarking with recent literature” provides an overall discussion; Section “Policy relevance, future work, and applicability” provides a brief policy relevance for Egypt and Section “Conclusions” concludes the study with main findings and future recommendations.

Egypt’s power sector has undergone a significant transformation, driven by grid modernization and smart technologies^14,15^. The country possesses some of the highest solar irradiance levels in North Africa, reaching 8 kWh/m^2^ ^16,17^. Projects like Benban Solar Park (4.5 GW) and the wind farms along the Red Sea corridor^18–20^ demonstrate Egypt’s technical readiness for large-scale hybrid systems. Studies on the Gulf of Suez, including the Zafarana feasibility reports^21–23^, confirm stable and high-quality wind regimes suitable for utility-scale deployment^24,25^.

## Literature review

### Global and national renewable energy trends

Recent studies highlight the global shift toward smart grids and hybrid systems to accommodate variable renewable energy^14,15^. Egypt has significantly expanded its renewable infrastructure, aiming for a 42% clean energy share by 2035^5^. The development of large-scale projects, such as the 4.5 GW Benban Solar Park and various wind farms along the Red Sea corridor^19^, demonstrates the country’s technical readiness for utility-scale hybrid integration. Moreover, the deployment of advanced storage technologies, including the 2400 MW Gebel Attaqa pumped-storage project, is essential for maintaining grid stability and long-term energy security^26,27^.

### Hybrid systems and studies on ras ghareb

Previous investigations have validated the feasibility of hybrid PV-wind systems in Egyptian coastal regions. Hamdi et al.^10^ and Ahmed^11,12^ evaluated the renewable potential in Ras Ghareb, confirming that its consistent wind speeds and high solar irradiance provide an optimal environment for energy-water infrastructure. However, existing literature often focuses on single-sector loads or lacks large-scale storage integration. This study addresses this research gap by modeling a mixed-sector load (residential, agricultural, and desalination) supported by a PV-Wind-PSH configuration, specifically tailored to the strategic needs of Ras Ghareb.

## Site description and resource potential

### Geographical context and rationale

Ras Ghareb is situated on the western shore of the Gulf of Suez at coordinates 28.35° N and 33.07° E. The city serves as a strategic industrial hub for oil exploration and supporting services, with a population of approximately 100,000 residents^15,28,29^. The site was selected for this study due to the critical water-energy nexus challenge it faces. In the absence of permanent surface water sources, seawater desalination, particularly Reverse Osmosis (RO), is the primary method for meeting potable water demands^30^. However, RO desalination is an energy-intensive process that requires a stable and sustainable power supply to be economically viable^2^.

### Solar and wind resource assessment

The region is characterized by high renewable energy potential, which supports the deployment of hybrid systems. Based on long-term NASA meteorological data, the site receives an average daily solar irradiance of 5.74 kWh/m^2^ ^31^. Solar radiation peaks during the summer months, which aligns with the seasonal increase in cooling and water demands. Regarding wind resources, the Gulf of Suez corridor is recognized for its stable wind regimes. At the Ras Ghareb site, the annual average wind speed is recorded at 7.06 m/s at a standard hub height^10,32^. The wind profile exhibits low turbulence intensity and high consistency, providing a reliable basis for wind energy generation.

In this study, the satellite-derived and reanalysis meteorological datasets… were cross-validated against neighboring national and international benchmarks^33,34^. This ensures simulation inputs are consistent with the solar and wind regimes of the Gulf of Suez^22,35^.

### Integrated load requirements

To evaluate the proposed PV-Wind-PSH system, this study models the integrated energy demands of three key sectors in Ras Ghareb:

- Residential Sector: Power requirements for 5000 households.
- Agricultural Sector: Irrigation needs for 2000 acres of farmland.
- Industrial Sector: Operational energy for an RO desalination plant with a daily capacity of 80,000 m3.

By incorporating these diverse load profiles, the model provides a comprehensive analysis of how hybrid renewable systems can support coastal urban development and food security.

In this study, the satellite-derived and reanalysis meteorological datasets used for the Ras Ghareb site were cross-validated against neighboring national and international benchmarks, including the Global Solar Atlas, the Global Wind Atlas, and available records from neighboring meteorological stations. This validation process ensures that the data alignment remains within the standard bias margins accepted for high-level feasibility assessments^33,34^. Such cross-referencing confirms that the simulation inputs are consistent with the solar and wind regimes of the Gulf of Suez, as reported in the aforementioned literature^22,35^.

## Study site and research rationale

### Technical design rationale

**Fig. 1:**
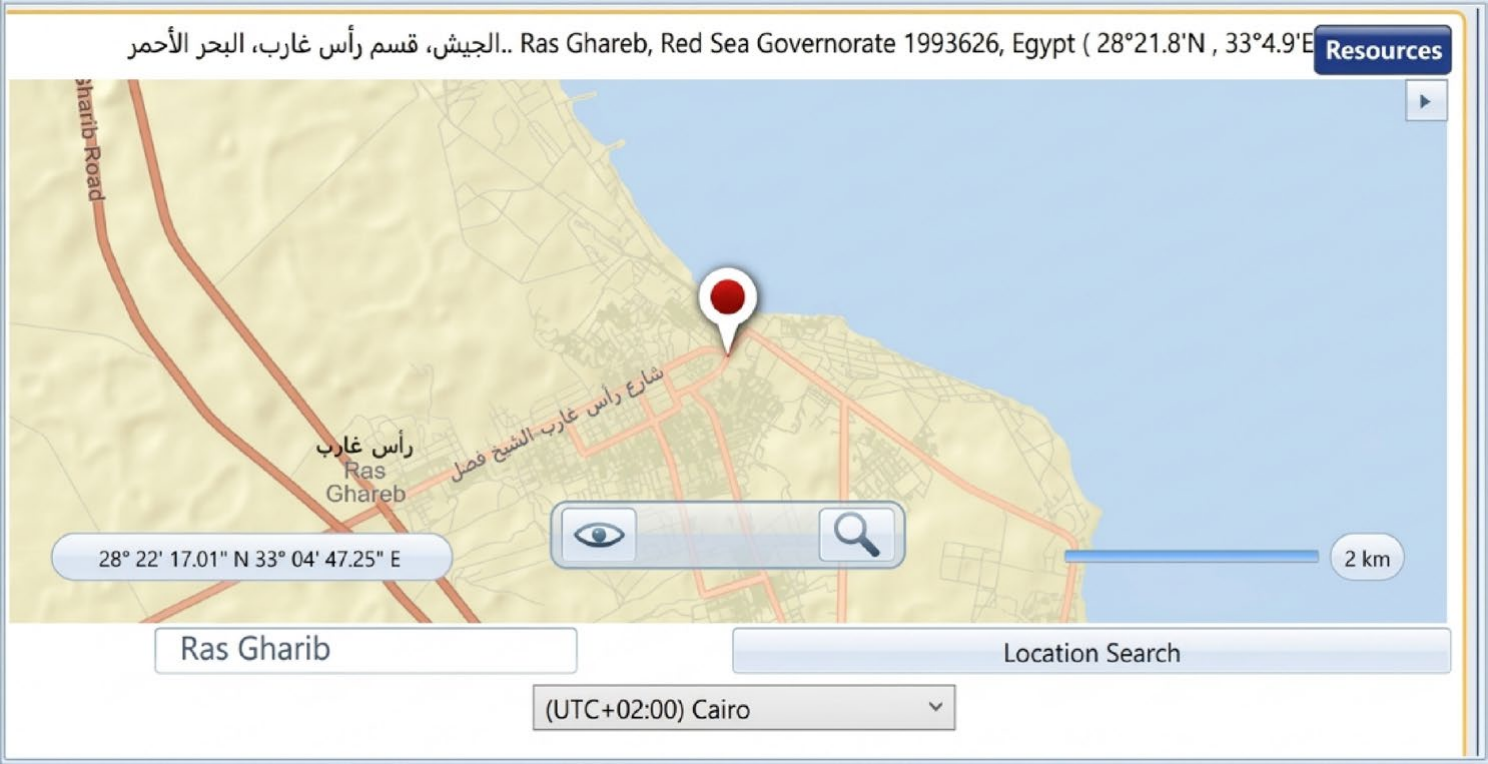
Road view image of the Ras Gharib site using Homer software.

Ras Ghareb, located along Egypt’s Red Sea coast, was chosen for this study due to its unique characteristics, which make it particularly suitable for hybrid renewable energy applications. Ezz et al.^30^ highlighted Ras Ghareb’s strategic position along the Red Sea, offering access to valuable groundwater resources, as well as an excellent potential for solar and wind energy generation. The region’s latitude of 28°21′33.9′′N and longitude of 33°4′30.5′′E, combined with its topographical and climatic conditions, make it ideal for deploying renewable energy systems. Figure 1 provides a map of Ras Ghareb’s location, visually illustrating its proximity to key energy resources and infrastructure.

Ras Ghareb’s geography and climate contribute significantly to its suitability for solar and wind energy generation. The area benefits from high solar irradiance, with NASA data confirming an average daily solar radiation of 5.74 kWh/m^2^ ^31^, positioning it among the highest in Egypt for solar energy production. Additionally, the region’s wind speeds, averaging 7.06 m/s as reported by Hamdi et al.^10^, provide a reliable resource for wind energy generation, reinforcing the potential for a hybrid solar-wind system. The combination of solar and wind resources creates an optimal environment for renewable energy deployment.

### Energy and water demand in Ras Ghareb

Ras Ghareb’s population of around 100,000 residents, along with its industrial expansion, particularly in oil extraction and supporting services, places increasing pressure on the local energy infrastructure. Abdel-Latif et al.^28,29^ noted that many remote coastal regions like Ras Ghareb face challenges related to water supply due to the lack of permanent freshwater sources. As a result, seawater desalination, especially through Reverse Osmosis (RO) technology, has become crucial for meeting water demands in these areas. However, as Shemer and Semiat^2^ highlighted, desalination is a highly energy-intensive process, necessitating efficient and sustainable energy solutions to support these operations.

This study addresses the need for a hybrid energy solution by integrating renewable energy sources to meet the electricity and water needs of Ras Ghareb. The system model incorporates the power requirements of 5000 residential homes, irrigation for 2000 acres of farmland, and the energy needs of an RO desalination plant producing 80,000 m^3^ of freshwater per day. This approach ensures that the energy demands of all key sectors are considered in the system design, offering a comprehensive and sustainable solution.

The methodology for estimating this water demand is summarized in Table 1. The net daily requirement for 5000 houses and 2000 acres of irrigation is 39,795 m3/day. To ensure continuous supply during cloudy days, a safety factor of 2 was applied, resulting in a total design capacity of approximately 80,000 m3/day. This necessitates a seawater intake of 160,000 m3/day based on a 50% recovery rate^15,36^.

**Table 1:** Summary of daily water demand components and desalination system design parameters for Ras Ghareb.

Water demand component	Estimated value
Domestic consumption (5000 Houses)	7255 m3/day
Irrigation requirement (2000 Acres)	32,540 m3/day
Total daily net requirement	39,795 m3/day
Reliability factor (safety margin)	2 day
Total design desalination capacity	~ 80,000 m3/day
Seawater intake (50% recovery rate)	160,000 m3/day

To provide a realistic simulation, the total electrical demand in Ras Ghareb is categorized into functional groups. As detailed in Table 2. The desalination plant is modeled as a 24/7 constant primary load to maintain operational stability. In contrast, irrigation loads are strategically managed as deferrable loads to maximize the utilization of daytime solar energy. The residential demand for 5000 households follows standard daily activity patterns. These hourly load profiles are integrated into the HOMER Pro model to ensure accurate techno-economic optimization and to reflect the system’s flexibility in managing surplus energy via the PHS system.

**Table 2:** Comprehensive breakdown of daily and annual electrical load categories.

Operational profile	Annual consumption (GWh/y)	Daily consumption (kWh/d)	Peak capacity (kW)	Load category
Constant/baseload (24/7)	185.7	508,800	21,200	Desalination plant
Deferrable (flexible)	239.2	655,286	27,304	Irrigation, agriculture
Diurnal Variation (primary)	17.7	48,480	2,020	Residential (5000 Houses)
Dynamic storage load	262.8	720,000	223,661	PHS storage
Integrated load profile	705.4	1,932,566	274,185	Total system load

The PHS capacity is based on the nominal storage capacity from HOMER Pro results. All daily and annual totals are calculated to align with the 705.4 GWh/yr total system demand.

### Rationale for hybrid system integration

Hybrid systems combining solar, wind, and storage technologies have proven effective in enhancing system reliability and reducing costs in remote areas. Ayadi et al.^14^ demonstrated that integrating multiple renewable sources with storage enhances grid stability by providing a continuous energy supply, even with intermittent resources. Lashin and Shata^35^ also noted that combining solar and wind resources in hybrid systems can optimize energy production, as their generation profiles complement each other.

However, the variability of solar and wind generation requires the inclusion of reliable storage systems to maintain supply during low-generation periods. Pumped-hydro storage (PSHPP) has been shown to provide a large-scale energy storage solution that balances supply and demand effectively. Abubakr et al.^26^ and Abubakr et al.^27^ emphasized the critical role of pumped-hydro storage in ensuring grid stability and long-term energy security, especially in areas with high renewable penetration.

In the case of Ras Ghareb, integrating pumped-hydro storage with solar and wind energy can provide a continuous power supply, mitigate intermittency, and reduce reliance on conventional power sources. The goal of this research is to investigate whether such a hybrid system can deliver a reliable, cost-effective, and environmentally sustainable energy solution for Ras Ghareb. To achieve this, the system is simulated and optimized using HOMER Pro, which enables detailed performance analysis and optimization under varying conditions.

To provide a transparent basis for the simulation and ensure reproducibility, the detailed technical specifications, search space, and configurations for each integrated component are presented and discussed in Section “Study methodology” (Table 3).

**Table 3:** Technical specifications and configurations of the system components.

Component	Model/type	Capacity/rating	Key technical parameters
Wind turbine	Enercon E-126	7.5 MW (22 Units)	Total capacity: 165,000 kW
Solar PV	M395-B1F	157,641 kW	Flat-plate PV
System converter	Eaton Power Xpert	114,000 kW	Dedicated for DC/AC conversion
Pumped hydro (PSHPP)	Ras Gharib PSHPP	223,661 kWh	880 strings
Grid connection	Central grid	999,999 kW	Net-metering enabled

## Study methodology

This study utilizes HOMER Pro software to model and simulate the integrated renewable energy system for Ras Ghareb. The tool is selected for its capability to evaluate various energy configurations through hourly energy-balance simulations^37,38^. While HOMER is used for techno-economic optimization, it is an energy-based tool. To ensure the model reflects physical reality, the simulation parameters were defined based on the actual 500 kV^19,39^. Transmission infrastructure from reliable regional data, and the grid interconnection limit was set within the software to 999,999 kW. This ensures that the electricity export levels are consistent with the technical capacity of the local grid and the Egyptian regulatory framework.

By incorporating detailed resource and demand data, the software generates and ranks different system configurations based on key performance indicators, such as cost, system reliability, and renewable energy contribution^38,40^.

### System modeling and methodology

The modeling workflow begins with assembling site-specific inputs: hourly solar irradiance and wind-speed time series, detailed load profiles for residential, agricultural and desalination demands, and techno-economic parameters for candidate components. Mas’ud^37^ demonstrated the value of this input-driven approach when applying simulation tools to assess hybrid systems for rural electrification, highlighting that realistic resource and load data are essential for credible sizing and cost estimation. Jimenez^38^ discussed how exploring multiple configuration options early in the design phase—varying generator sizes, storage capacities and operational rules—reduces the risk of selecting suboptimal architectures. Murthy et al.^41^ emphasized that component-level modeling must include operational constraints (e.g., inverter limits, converter behavior, and control strategies) because these affect both reliability and dynamic interactions between renewables and storage. Afif et al.^42^ further showed through case studies that combining detailed techno-economic inputs with systematic configuration screening produces robust optimal designs for standalone and grid-connected hybrid plants.

Following data assembly, the study simulates a broad set of candidate configurations and evaluates each against technical and economic metrics (supply reliability, Net Present Cost, payback period, and renewable fraction). Component lifetimes, fixed and variable O&M, replacement costs, and discount/inflation rates are included in the cash-flow model so that ranking reflects realistic life-cycle economics. Sensitivity runs then test how key uncertainties (resource variability, component costs, project lifetime) affect the preferred design, ensuring the selected configuration remains robust under plausible futures.

To mathematically evaluate the system’s performance and ensure an optimal techno-economic design, the optimization process is governed by a multi-objective framework. The primary objective is the minimization of the Total Net Present Cost (NPC), which represents the life-cycle cost of the hybrid plant, including capital, replacement, and O&M costs. Additionally, the study aims to minimize the Levelized Cost of Energy (LCOE) and reduce greenhouse gas emissions to enhance environmental sustainability. These objectives are achieved while maintaining 100% system reliability (zero capacity shortage) to meet the integrated demands.

The economic performance is evaluated using the following formulations as implemented in the HOMER Pro to determine the hybrid system’s net cost, which calculates the system’s total cost over its lifetime and then subtracts the incurred revenue. The total net present cost (NPC) is calculated using the following equation^43^:

1 $${\mathrm{C}}_{{{\mathrm{NPC}}}} = \frac{{{\mathrm{C}}_{{{\mathrm{ann}}.{\text{ tot}}}} }}{{{\text{CRF }}\left( {{\mathrm{i}}.{\mathrm{R}}_{{{\mathrm{proj}}}} { }} \right)}}$$

where $${\mathrm{C}}_{\mathrm{a}\mathrm{n}\mathrm{n}. \mathrm{t}\mathrm{o}\mathrm{t}}$$ is the total annualized cost ($/yr.), $$i$$ is the annual real interest rate (the discount rate), Rproj is the project lifetime, and CRF is the capital recovery factor, given by the equation^44,45^:

2 $${\mathrm{CRF}}\left( {i.{\mathrm{N}}} \right) = \frac{{i \left( {1 + i} \right)^{N} }}{{\left( {1 + i} \right)^{N } - 1}}$$

where i is the annual real interest, rate and N is the number of years. HOMER uses the following equation to calculate the levelized cost of energy:

The LCOE is equally a significant component of the system design and it indicates how economically profitable the system can be. It is calculated using the following equation^44,45^:

3 $${\mathrm{LCOE}} = \frac{{{\mathrm{NPC}}}}{{\mathop \sum \nolimits_{{{\mathrm{t}} = 1}}^{{{\mathrm{t}} = 8760}} {\uprho}_{{{\mathrm{load}}}} }} \times {\mathrm{CRF}}$$

where *P*_lead_ is the load power.

To reflect the non-simultaneous peak power consumption of the integrated residential, agricultural, and desalination sectors, the peak load demand was adjusted using a Diversity Factor (DF), as supported by literature^46–48^. The diversified peak load (${P}_{div}$) is defined by the following equation:

4 $$P_{div} = \frac{{P_{i. max} }}{DF}$$

where ${P}_{i. max}$ represents the sum of individual maximum demands of the various sectors.

Diversity factor ≥ 1.

Group Diversity factor ≥ 1.1^49^

In this study, a DF of 1.3 was applied for individual residential units, while a Group Diversity Factor of 1.1 was utilized for the total system load. This approach is consistent with industry standards for hybrid system optimization, ensuring that the generation capacities are technically justified and not unnecessarily over-sized.

To ensure the system’s reliability and economic feasibility, the HOMER Optimizer was configured with a broad search space for each component. Given the substantial total daily load of 1,212,593 kWh/day.

To ensure the system’s reliability and economic feasibility, the HOMER Optimizer was configured with a broad search space for each component. Given the substantial total daily load of 1,212,593 kWh/day, the search space for PV and wind capacities was defined to cover up to 100% of this demand. This allowed the software to identify the optimal sizing of 157,640 kW PV and 22 wind turbines required to achieve a 0.00% capacity shortage. Additionally, the converter capacity was set at 206,000 kW to handle peak instantaneous production, while the PSHP reservoir was optimized (up to 1,109,376 m^3^) to match the reversible turbines’ requirements and ensure continuous supply during periods of resource intermittency. This systematic sizing approach ensures that the large-scale capacities reported are technically justified by the combined energy-water-food nexus demands.

### Hybrid system components

The hybrid energy system considered in this study consists of photovoltaic (PV) generation, wind turbines, pumped-hydro storage (PHS), and an optional grid connection. Each component is modeled using technical parameters obtained from manufacturer data sheets and values commonly reported in the literature. The PV arrays were optimized to a capacity of 157.6 MW to meet the massive daily energy requirements of the residential, agricultural, and desalination sectors. It is essential to clarify that the PHS system functions as a closed-loop energy storage medium and is not used for direct water supply. The desalination plant is the sole provider for all site requirements (drinking, irrigation, and industry). The site’s coastal topography, characterized by significant elevation differentials near the Red Sea, provides the necessary natural head for the PSHP reservoirs^19,50^. Furthermore, to account for potential evaporation losses in the PHS reservoirs due to the arid climate of Ras Ghareb, the desalination plant is designed to provide ‘makeup water’ to maintain the required hydraulic levels^51^. Modeling the desalination load as a constant industrial demand is, therefore, a deliberate design choice to ensure that the system can simultaneously meet the maximum community water demand and maintain the energy storage integrity under all operational conditions. Similarly, a total of 22 wind turbines (totaling 166.1 MW) were selected based on their power curves and the measured wind-speed distribution in Ras Ghareb, ensuring that turbine performance is representative of the site’s high-wind coastal environment. The PHS unit is characterized by its reservoir volume, hydraulic head, round-trip efficiency, and operating constraints, enabling the storage of excess renewable energy and dispatch during low-generation periods. Where applicable, a grid interface is included to allow both import during deficits and export during times of surplus generation.

### PSHP operational strategy and physical constraints

The PSHP system, utilizing Reversible Francis Turbines, serves as a critical balancing component within the hybrid plant. Its operational logic is specifically designed to manage the intermittent nature of renewables: pumping occurs during daylight hours to store energy when solar PV production is at its peak, while generation is activated during the night or when wind speeds drop below the required threshold. This ensures a consistent supply to meet the 30,230 kW demand. To account for hydraulic losses, a round-trip efficiency $(\eta )$ of 90% was applied. The system follows the Cycle Charging (CC) dispatch strategy, prioritizing the reservoir’s state-of-charge to ensure it acts as a reliable ‘green battery’ that bridges the gap between solar-heavy daytime production and nighttime consumption, all while remaining within the physical constraints of the 1,109,376 m3 reservoir and 200 m head.

### Optimization and performance criteria

Each simulated configuration is evaluated using a standardized set of technical and economic indicators. The Net Present Cost (NPC) quantifies the lifetime cost of the system, incorporating capital investment, component replacements, operations and maintenance, and salvage value. The Cost of Energy (COE) is computed to assess the unit cost of delivered electricity, allowing comparison between scenarios with different capital intensities. To ensure the reproducibility and verification of these economic metrics, the detailed cost parameters—including capital, replacement, operations and maintenance (O&M) costs, as well as the discount rate and project lifetime—are summarized in Table 3.

The renewable fraction measures the contribution of PV, wind, and PHS to the total energy supply and serves as a key sustainability metric. Additional performance indicators include the annual unmet load, storage utilization, and excess electricity fraction. These metrics enable the identification of configurations that balance economic feasibility with technical reliability and environmental performance.

### Sensitivity analysis approach

A structured sensitivity analysis is conducted to assess the robustness of the system performance under varying assumptions. Key uncertain parameters—including future component prices, discount rate, and grid export tariffs—are varied across plausible ranges. It is important to clarify that the proposed system for Ras Ghareb does not incorporate fossil fuel-based backup (e.g., diesel generators); therefore, fuel price sensitivity is not applicable. Instead of applying artificial resource fluctuations (e.g., ± 10%), this study utilizes 30 years of historical wind data and 22 years of solar irradiance data from NASA POWER^6,23,52^. These extensive datasets inherently capture the actual seasonal and inter-annual variability specific to the region, ensuring that the system’s resilience is validated against realistic long-term climatic fluctuations.

Although the system optimization was performed under baseline economic and resource conditions, it is useful to consider how variations in key parameters could influence the performance of the hybrid configuration. The following qualitative sensitivity analysis provides a conceptual evaluation of potential changes without requiring additional simulations.

- Variation in PV capital cost:

A reduction in PV capital cost, such as the 10–20% decline reported in recent global trends^53,54^, would further improve the economic performance of the system by lowering the Net Present Cost and increasing profitability. Since the hybrid configuration already relies on substantial PV generation, cost reductions would strengthen the competitiveness of the proposed design.

- Variation in annual wind speed:

A decrease in average wind speed would reduce annual wind energy production, affecting the system’s energy balance given the large contribution of wind (over 60%) in the baseline scenario. In this case, the pumped-hydro storage unit would experience more frequent discharge cycles, and grid imports may increase slightly. However, because wind speeds in Ras Ghareb are consistently high^55^, the system is expected to remain viable under moderate variations.

- Variation in grid export price:

The hybrid system exhibits strong profitability due to revenues from selling a substantial annual surplus of 520.89 GWh/yr to the grid. Based on the $0.064/kWh^56^ sellback tariff (Feed-in Tariff) regulated by Egypt ERA, the system achieves its reported negative Net Present Cost (NPC). A reduction in the grid export tariff would decrease this revenue stream and reduce the magnitude of the negative NPC. Nonetheless, the system’s high renewable fraction, combined with the low subsidized purchase price of $0.024/kWh^57^, means that the economic feasibility would still surpass that of the grid-only alternative. This confirms that the system’s profitability is highly resilient to potential fluctuations in the national grid’s pricing framework.

- Variation in pumped-hydro storage capacity:

Increasing the storage capacity would enhance the system’s ability to manage seasonal fluctuations in wind and solar resources, potentially reducing grid imports further. Conversely, a smaller reservoir would increase system sensitivity to low-generation periods, particularly during winter months. However, due to the high renewable penetration at the site, moderate reductions in PSH capacity would not critically impact reliability.

Overall, these qualitative trends suggest that the proposed hybrid system remains technically and economically robust under reasonable variations in resource availability, technology cost, and grid pricing conditions.

### Simulation scenarios

Three principal scenario groups are analyzed to capture a comprehensive range of system behaviors:

1. Grid-only case:

Serves as the baseline scenario, representing electricity supplied solely from the national grid. It provides a benchmark for comparing economic and environmental benefits of hybrid alternatives.

2. Hybrid renewable configuration:

Evaluates PV-wind-PHS combinations sized to meet the full electricity demand of residential, agricultural, and desalination loads. This scenario identifies cost-effective designs capable of minimizing NPC and maximizing renewable contribution.

**Fig. 2:**
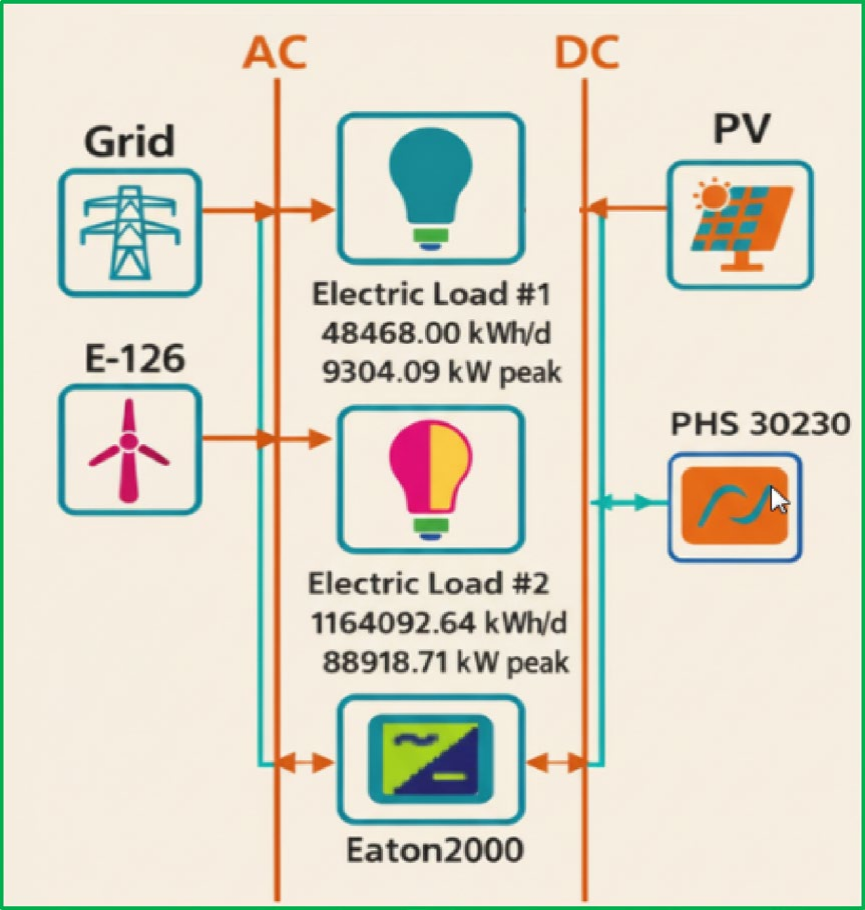
Grid-connected hybrid power system diagram.

3. Resource and cost sensitivity scenarios:

Explores system behavior under high-irradiance/low-irradiance and high-wind/low-wind years, as well as alternative capital cost assumptions for PV, wind turbines, and storage. These simulations test overall system adaptability and help determine parameter thresholds at which the hybrid system remains competitive (Fig. 2).

## Analysis of electrical loads

Understanding the nature of the electrical load is a crucial first step in designing an efficient and reliable hybrid energy system. In this research, two primary electrical loads were meticulously analyzed to determine the site’s essential energy requirements. Accurately.

### Electric load 1: residential load profile

This load represents the daily energy consumption of 5000 rural households in the area. The average daily consumption was carefully estimated based on the types of common household appliances and their typical operating hours, as detailed in Table 4^49,58^. This research moves beyond generalized load profiles to present a specific and realistic model that accurately reflects the consumption behavior of the local community.

**Table 4:** Average daily consumption of household appliances.

Electrical loads	Power (w)	Quantity	Number of operating hours(h/day()	Electrical load capacity (w)	Energy consumed per day (Wh/d)
Washing machine	200	1	1	200	200
Refrigerator	200	1	24	200	4800
TV + receiver	35	1	8	35	280
Fan	100	3	12	300	3600
Blender	400	1	1	400	400
LED lights	9	6	12	54	648
Handheld clothes iron	1000	1	0.25	1000	250
Radio	10	1	12	10	120
LED lights	24	3	12	72	864
Computer	450	1	6	450	2700
Total				2721	13,862

**Fig. 3:**
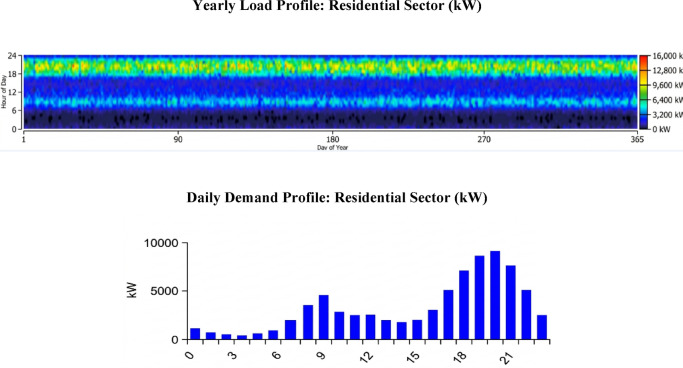
Average demand for residential loads “electrical load No. 1” in the Ras Ghareb area of Egypt.

Figure 3 provides a detailed analysis of Electric.

Load #1, which represents the household consumption profile for the proposed system. This analysis is fundamental to accurately sizing the system’s components and ensuring an efficient, reliable power supply.

The daily consumption pattern for this load shows a gradual increase throughout the day, peaking at 9304.09 kW in the evening hours, specifically between 6:00 PM and 9:00 PM. Based on the input data, the average daily consumption is 48,468.00 kWh. This load profile has a low load factor of 0.22, indicating a highly volatile load with sharp, pronounced peaks that require a robust system design capable of handling abrupt demand fluctuations.

### Electric load 2: industrial load profile (agricultural & water)

**Fig. 4:**
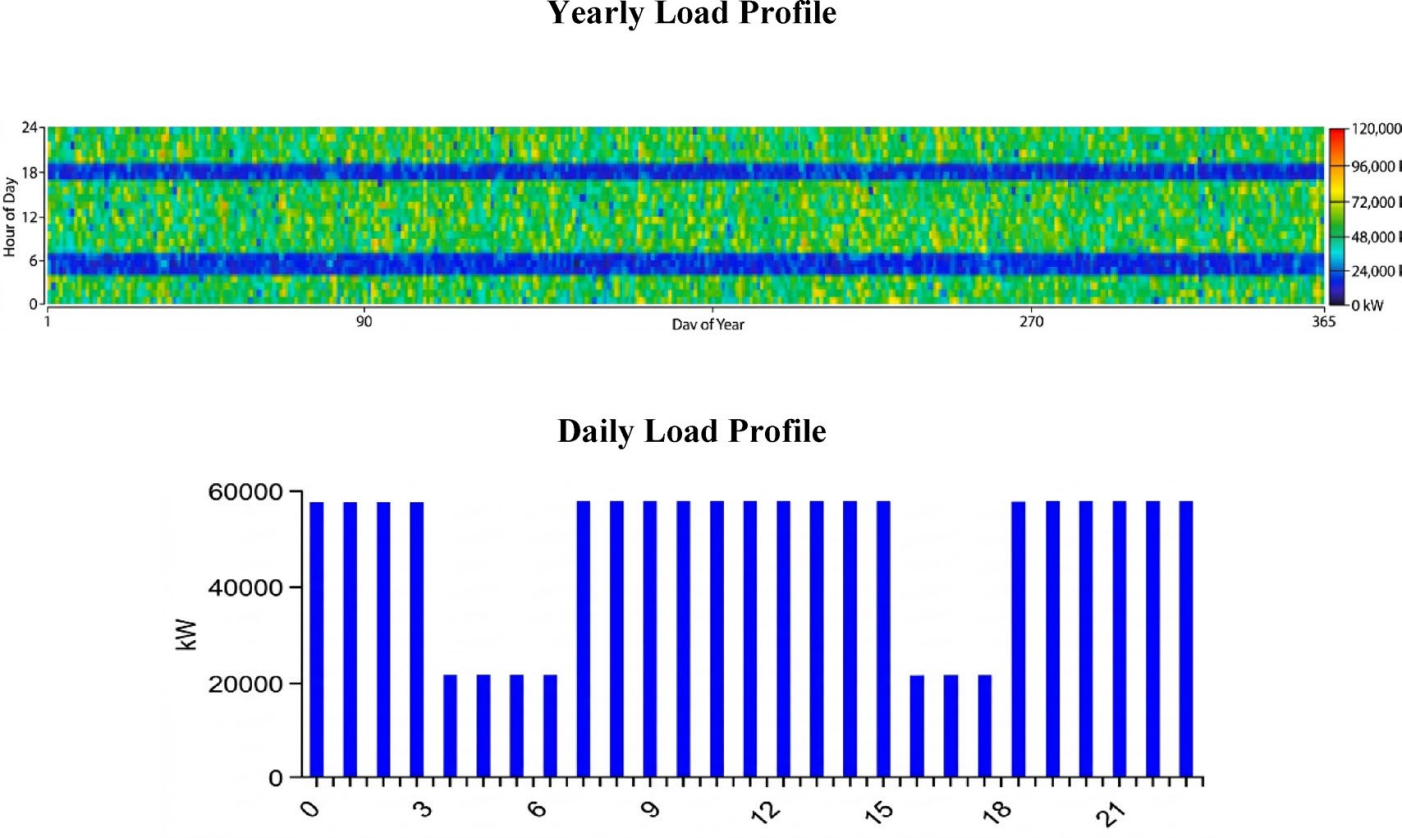
Average demand for industrial load “Electrical Load No. 2” in the Ras Ghareb area in Egypt.

Figure 4 provides a detailed analysis of the Electric Load #2,

This industrial load profile differs from the residential one, and is characterized by high continuous energy consumption. It represents the combined power demand of key on-site agricultural operations, including irrigation systems for 2000 acres of agricultural land, the energy-intensive reverse osmosis desalination plant, and the pumping requirements of the pumped-hydro storage system.

The daily profile of this load is remarkably constant and high throughout the 24-h cycle, reflecting the nonstop nature of these industrial processes that require a continuous energy supply. This massive load confirms its status as the system’s largest energy consumer. The average daily consumption is 1,164,092.64 kWh/day, with a peak load of 100,318.47 kW. The load is further characterized by a load factor of 0.48, indicating a more stable and regular demand profile, which is beneficial for the system’s operational efficiency.

### Conclusion of load analysis

This analysis reveals that the two electrical loads (residential and industrial) exhibit significantly different characteristics. The residential load requires handling sharp evening peaks, while the industrial load demands a large, continuous supply. These data formed the basis for the HOMER software’s design of the proposed hybrid system, ensuring it can efficiently meet all requirements. The system leverages pumped-hydro energy storage to compensate for the natural fluctuations of the renewable energy sources (solar and wind).

## Hybrid system resources

### Solar resources

Analyzing the solar radiation resources at the proposed site is a key step in ensuring the effectiveness of the photovoltaic system. Weather data for PV were obtained using the PVGIS database^33^, providing a reliable record of solar potential. To ensure climatic representativeness, the solar irradiance data covers a 22-year period (1983–2005) of satellite-derived monthly averages, ensuring that the interannual variability of solar resources is well-captured.

**Fig. 5:**
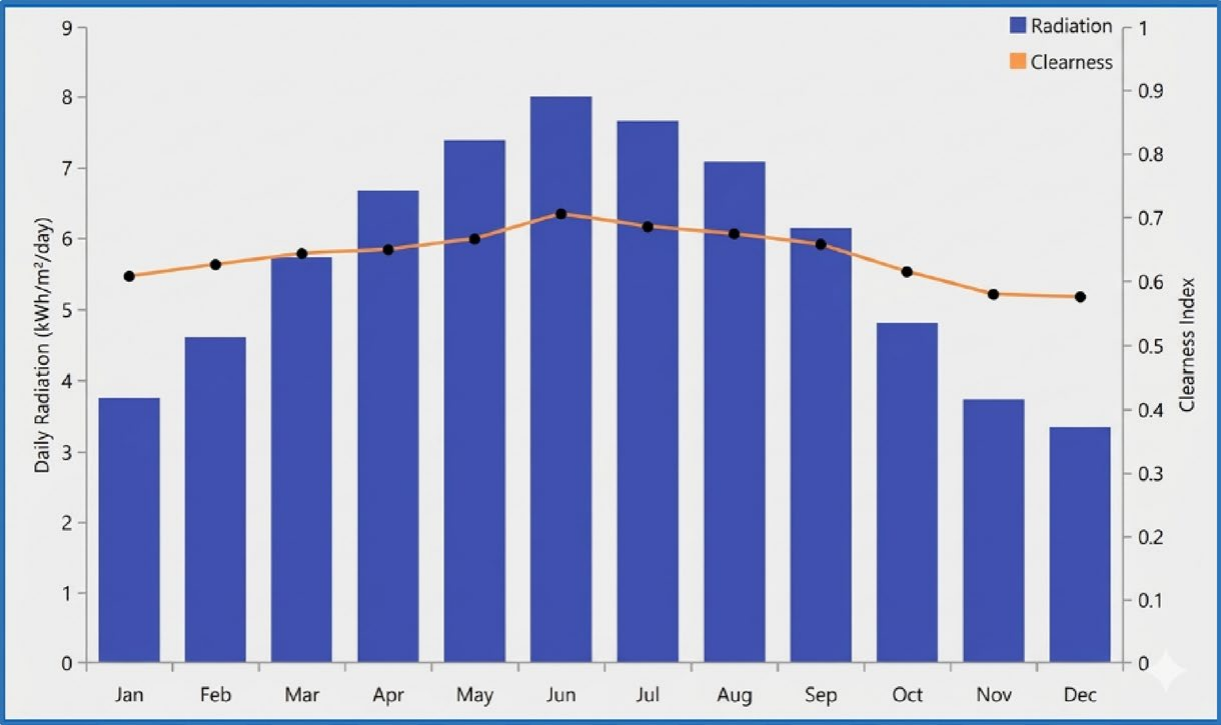
Monthly solar radiation and clearness index.

Figure 5 illustrates the monthly profile of the average solar irradiance at the site, where the annual average is 5.74 kWh/m2 per day. An analysis of the data reveals the following:

- *Monthly peak* Solar irradiance reaches its highest levels in May, June, and July, with the average irradiance peaking at approximately 8.010 kWh/m2 in June.
- *Monthly lows* The irradiance gradually decreases during the winter months, reaching its lowest levels in December and January, with averages of 3.330 kWh/m2 and 3.750 kWh/m2, respectively.
- *Clearness index* The clearness index peaks at 0.705 in June and drops to its lowest value of 0.574 in December. These values indicate a generally clear sky throughout the year with high solar potential.

These data confirms that the proposed site has excellent year-round solar potential, making it an ideal location for solar energy generation.

### Wind resources data analysis

Evaluating wind resources at a proposed site is a crucial step in determining the viability of a wind power project. Data on average wind speed in the Gulf of Suez region were obtained from the Egyptian Wind Atlas^33^.

- *High-Potential Site* Ras Ghareb is considered to have very high wind energy potential. Statistical studies show that the Ras Ghareb area has the second-highest wind density after Gabal El-Zeit, with average wind speeds of 9.8 m/s at 24.5 m above ground level and 12.5 m/s at 100 m^33^.
- *High Wind Power Density* Previous studies have also clearly shown a high wind power density of approximately 900 kW/m2 annually at a height of 100 m in the Ras Ghareb area^23^.

The Egyptian Wind Atlas^33^ confirms the high potential for wind power generation in Egypt and the Gulf of Suez region.

**Fig. 6:**
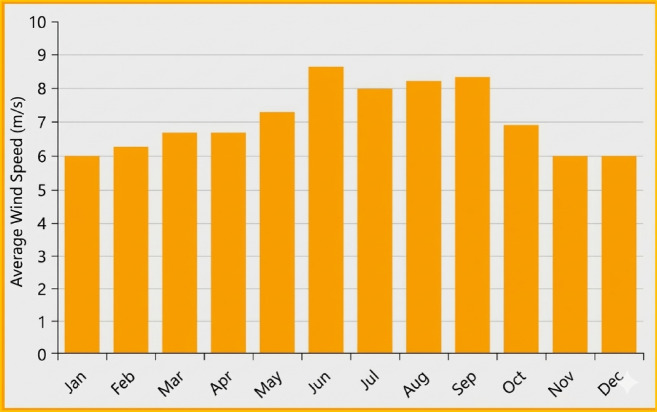
Average wind speed during the months of the year.

Figure 6 shows the annual wind speed profile at the site, with an annual average wind speed of 7.06 m/s. This profile is based on a 30-year climatological average (1984–2013) sourced from the NASA POWER database and cross-referenced with the Egyptian Wind Atlas^33^. Using such a long-term data span (30 years) enhances the reliability of the techno-economic optimization by accounting for long-term wind speed fluctuations.

A detailed analysis of the monthly data reveals the following:

- *Monthly Peak* Wind speeds reach their highest levels during June, July, August, and September, with the average in July being approximately 7.960 m/s, in August 8.210 m/s, and in September 8.330 m/s.
- *Monthly Lows* Wind speeds decrease significantly during the winter months, reaching their lowest levels in January and February, averaging 5.970 m/s and 6.220 m/s, respectively.

### Pumped hydro storage (PHS) economic modeling

The economic modeling of the Pumped Hydro Storage (PHS) system is based on verified international benchmarks. According to established literature^59^, the capital cost for large-scale PHS projects typically ranges from $1050 to $7650 per kW. For this study, a conservative value of $3500/kW was adopted to ensure a realistic economic assessment. Since HOMER Pro requires cost inputs based on reservoir capacity (m3), the capacity-based costs were converted as shown in Table 5. This ensures that the simulation accurately reflects the hydraulic and mechanical scale of the Ras Ghareb site while maintaining economic transparency.

**Table 5:** PSHPP Cost Parameters and HOMER Pro Input Conversion.

Parameter	Value
Designed pump capacity	76,490.27 kW
Unit capital cost	3500 $/kW
Total capital investment	267,715,945 $
Annual O&M (1% of Capital)	2,677,159.45 $/yr
Reservoir capacity	1,109,376 m3
HOMER capital input	241.32 $ / m3
HOMER O&M input	2.41 $ / m3 / yr

## Hybrid system schematic

**Fig. 7:**
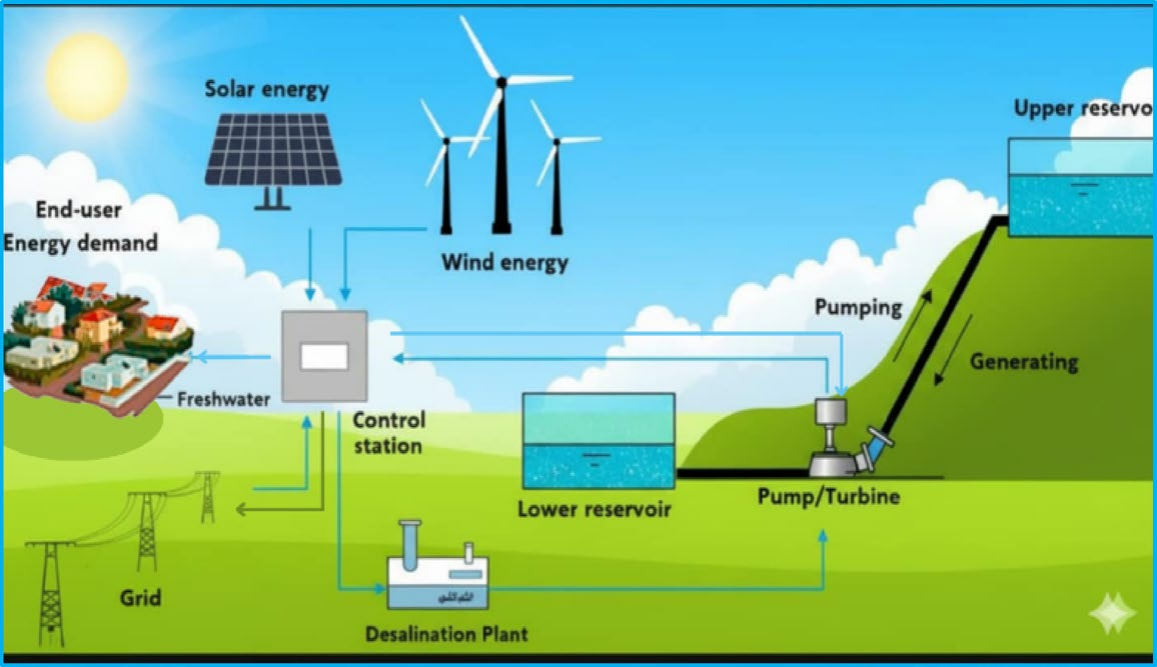
Hybrid system schematic.

Figure 7 presents the schematic diagram of the proposed hybrid system. This diagram is a crucial preliminary step, as it clearly illustrates the system’s basic components and their interconnections before the simulation and optimization process begins.

The schematic highlights the system’s architecture, showing how multiple energy sources are integrated to meet various loads efficiently. It serves as a visual roadmap that ensures the accurate setup of all components, enabling a detailed analysis of the system’s energy flow and operational relationships.

The system requires an inverter to convert the Direct Current (DC) generated by the Photovoltaic (PV) array into Alternating Current (AC) for integration with the wind turbine output and to supply the AC loads, including the energy-intensive desalination plant.

The primary components of the hybrid system are:

- Photovoltaic (PV) array
- Inverter
- E-126 wind turbine
- Reverse Osmosis (RO) desalination plant
- Pumped-Hydro Storage (PSH)
- Upper and lower water reservoirs/tanks
- Pumps / Reverse Francis Turbines (for Pumped-Hydro Storage operation)
- Public electricity grid (connection to the Egyptian national electricity grid to allow for energy exchange)

## Modeling and simulation of the hybrid solar / wind system components

Optimization Framework and Multi-Objective Trade-offs:

The optimization process in this study was governed by a structured hierarchy to resolve trade-offs between technical reliability, environmental impact, and economic feasibility. First, the Technical Objective was set as a non-negotiable constraint, requiring 100% reliability for the desalination plant; any configuration with a capacity shortage was excluded. Second, the Environmental Objective prioritized maximizing renewable penetration, resulting in a system that acts as a carbon sink for the national grid. Finally, the Economic Objective focused on selecting the architecture with the lowest Net Present Cost (NPC) among the technically and environmentally feasible solutions. The trade-off between the high capital investment of PSHPP and lower-cost grid alternatives was resolved through long-term lifecycle analysis. While the hybrid system involves higher initial costs, its ability to reduce annual operating expenses and generate grid revenue resulted in a superior Internal Rate of Return (53%), justifying its selection over intermediate alternatives.

The HOMER software is an effective tool for modeling and analyzing the performance of integrated hybrid energy systems. It applies specific methodologies to calculate economic and technical performance, as detailed below:

The mathematical framework governing the system components is detailed below to ensure scientific transparency:

(a) PV Array Model: The power output (PPV) is calculated based on solar irradiance and temperature effects^34^.

5 $${\mathrm{P}}_{{\text{PV }}} = {\mathrm{H}}_{{{\mathrm{PV}}}} \times {\mathrm{U}}_{{{\mathrm{PV}}}} \left( {\frac{{{\mathrm{V}}_{{\mathrm{T}}} }}{{{\mathrm{V}}_{{{\mathrm{T}}{\mathrm{.STC}}}} }}} \right)\left[ {1 + \beta_{{{\mathrm{P}}\left( {{\mathrm{T}}_{{{\mathrm{C}} }} - {\mathrm{T}}_{{{\mathrm{C}}{\mathrm{.STC}}}} } \right)}} } \right]$$

where:

${H}_{PV}$: The PV array power capacity in kilowatts (kW).

${H}_{PV}$: represents the PV array power capacity in kW.

${U}_{PV}$: The PV array derating factor.

${V}_{T}$: The incident solar radiation on the PV array in kW/m2.

${V}_{T.STC}$: The solar radiation under standard test conditions.

${\beta}_{P}$: The temperature coefficient based on power.

${T}_{C}$: The cell temperature.

${T}_{C.STC}$: The cell temperature under standard temperature conditions.

The ambient temperature data for the Ras Gharib site were sourced from the NASA POWER database, with an observed annual average of 22.89°C. These hourly temperature variations are crucial for determining the cell temperature (TC) in Equation (1). The model accounts for thermal losses by adjusting the PV power output based on the temperature coefficient (βP), ensuring that the simulation reflects the real environmental conditions of the study area, particularly during summer months when higher temperatures can impact PV performance.

(b) *Wind speed* is measured using the power law at heights ranging from 127 to 135 meters, applying the following equation^34^:

6 $${{{\mathrm{W2}}} \mathord{\left/ {\vphantom {{{\mathrm{W2}}} {{\mathrm{W1}}}}} \right. \kern-0pt} {{\mathrm{W1}}}} = \left( {{{{\mathrm{Y2}}} \mathord{\left/ {\vphantom {{{\mathrm{Y2}}} {{\mathrm{Y1}}}}} \right. \kern-0pt} {{\mathrm{Y1}}}}} \right)\alpha$$

where:

$$W2: The wind speed at height Y2.$$

W1: The wind speed at height Y1.

α: The power law exponent.

Second, the power produced (${P}_{W}$) is modeled via the cubic relationship with wind speed^60^:

7 $$P_{W} = \frac{1}{2} \rho AV^{3} C_{\rho } \left( {\lambda \beta } \right)$$

where $\rho$ is the air density (kg/m3), A is the rotor swept area (m2), v is the calculated wind speed (m/s), and Cp is the power coefficient representing the turbine’s efficiency. The total farm output (${E}_{F}$) is then determined by the number of units (${N}_{T}$) as^46^:

8 $$E_{F} = P_{WT} \times N_{WT}$$

(c) The energy stored by the pumped-storage hydropower (PSH) plant is calculated using the following equation. This is the standard equation adopted by HOMER for modeling, as it converts the water’s potential energy into electrical units^61^:

9 $$E_{{{\mathrm{SP}}}} = {9}.{81} \cdot \eta \cdot {\mathrm{H}} \cdot {{{\mathrm{Vol}}} \mathord{\left/ {\vphantom {{{\mathrm{Vol}}} {{36}00}}} \right. \kern-0pt} {{36}00}}$$

where:

${E}_{\mathrm{S}\mathrm{P}}$: The energy stored by the PSH plant in kWh.

η: The plant’s efficiency (%).

H: The available head in meters (m).

Vol: The reservoir’s volume in cubic meters (m3) (Table 6).

**Table 6:** Storage system features.

Parameter	Symbol	Value
Reservoir size	Vol	1,109,376 m3
Available head	H	200 m
Flow rate (turbine)	Q	17.12 m3/s
Efficiency	η	90%
Power	P	30,230 kW
Energy stored	ESP	544,148.93 kWh

(d) *Desalination Power Demand*: Calculating the energy required daily for a seawater desalination plant^49,62^.

(DP_RO_) for the RO system is:

10 $${\mathrm{DP}}_{{{\mathrm{RO}}}} = {\mathrm{D}}_{{{\mathrm{FW}}}} \times \gamma$$

where:

- DP_RO_ is the power delivered to the RO system MW h / d
- D_FW_ is the freshwater demand m3 / d

γ is the unit RO power consumption factor kWh/m3.

(e) *Grid Connection and Economic Interaction* In the grid-tied scenario, the utility grid functions as both a source and a sink. HOMER models this interaction by balancing the grid purchase price (Cp,x in $/kWh) and the grid sell-back price (Csb,x in $/kWh). The net annual cost (Cge) is derived by calculating the difference between the total energy purchased (Egp,x,y) and the revenues generated from sold energy (Egp,x,y) across different rates (x) and months (y)^39,60^:

11 $$C_{ge} = \mathop \sum \limits_{ \times } \mathop \sum \limits_{\gamma } \left( {E_{gp,x,y} \times C_{{P{,}X}} } \right) - \mathop \sum \limits_{ \times } \mathop \sum \limits_{\gamma } \left( {E_{gp,x,y} \times C_{P,X} } \right)$$

### Analysis of PSHPP and desalination demand interaction

The interaction between the PSHPP and the desalination plant is fundamental to the system’s reliability. At the Ras Ghareb site, the simulation applies the Cycle Charging (CC) dispatch strategy. This operational logic establishes a structured hierarchy for energy distribution to ensure continuous water production and target storage levels. The control logic designates the desalination plant as a primary, non-interruptible load; during daylight hours, the solar and wind energy directly supply the desalination unit along with the residential and irrigation requirements. Simultaneously, the Francis turbines operate in pumping mode as a prioritized charging load, utilizing the available renewable energy to elevate water to the upper reservoir until it reaches its maximum capacity. To address grid hosting capacity and operational constraints, the simulation parameters were configured to reflect the robust infrastructure of the Ras Ghareb wind corridor. Specifically, the Ras Ghareb site was selected because it is interconnected with the Egyptian National Grid via ultra-high voltage (500 kV) transmission lines^19,40,62^, effectively eliminating transmission constraints for the capacities produced in this study. The grid interconnection limit was set at 1 GW (999,999 kW), ensuring that the high volume of energy export—totaling approximately 520 GWh/yr—is absorbed without curtailment. The PSHPP units (880 units) and the desalination plant function as dynamic internal sinks, smoothing power fluctuations before grid injection. Consequently, the system maintains a 0% capacity shortage and generates a significant negative O&M cost of − $553,033,533/yr, representing the annual revenue from surplus electricity sales under the Net Metering framework.

## Results analysis and ecnomic discussions

### Simulation results overview

This section provides a comprehensive summary of the final economic and operational results of the proposed hybrid renewable energy system compared to the baseline case (grid-only). To justify the selection of the final system architecture, a rigorous optimization process was conducted in HOMER Pro to evaluate various intermediate configurations. As summarized in Table 7, excluding the PV component (Wind/PHS/Grid) increases the NPC to $212 M, while a configuration relying solely on PV and storage (PV/PHS/Grid) results in an NPC of $514 M. Furthermore, configurations without storage (Wind/Grid) showed lower renewable penetration (82.0%) and higher energy costs ($0.0331/kWh). These results confirm that the integration of PV, Wind, and PHS is the optimal technical and economic solution for the Ras Ghareb site, achieving the lowest NPC (− $94.7 M) and a negative LCOE (− $0.00987/kWh).

**Table 7:** Comparison of alternative system architectures (HOMER optimization results).

System architecture	NPC ($)	LCOE ($/kWh)	Operating Cost ($/yr)	Ren. Fraction (%)
PV / Wind / PHS / Grid (Proposed)	− 94.7 M	− 0.00987	− 45.4 M	93.8
Wind / PHS / Grid (No PV)	212 M	0.0220	− 27.6 M	88.3
Wind / Grid (No Storage)	260 M	0.0331	4.60 M	82.0
PV / PHS / Grid (No Wind)	514 M	0.110	15.6 M	89.4

### Comprehensive economic analysis and profitability

The economic evaluation of the proposed hybrid system demonstrates robust financial performance, facilitating a strategic transition from a cost-intensive desalination facility to a revenue-generating asset. The results indicate that the integration of multi-source renewables creates a synergistic effect that optimizes both capital utilization and operational efficiency.

#### Interpretation of negative NPC and revenue streams

In this study, the Net Present Cost (NPC) is reported at a negative value of − $94.7 Million. As illustrated in the comparative analysis in Table 8, this reflects a superior financial standing compared to the base system (grid-only), which carries a total NPC of $1.41 Billion. This negative NPC signifies that the project’s cumulative revenues over its 25-year lifespan exceed the total costs of investment and operation. This outcome is primarily attributed to the substantial annual energy export of 520.89 GWh/yr to the national grid. Supported by a fixed sellback tariff of $0.064/kWh [160], the system effectively functions as a utility-scale power provider while simultaneously meeting local water and energy demands.

**Table 8:** Summary of the economic comparison results.

Metric	Base system	Proposed system
Total NPC	$1.41B	− $ 94.7 M
Initial CAPEX	$0.00	$358 M

Table 8 summarizes the economic comparison between the Base System and the Proposed System.

#### Capital recovery and investment indicators (IRR and ROI)

**Fig. 8:**
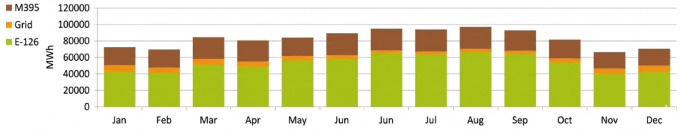
Monthly electrical energy production from different sources.

**Fig. 9:**
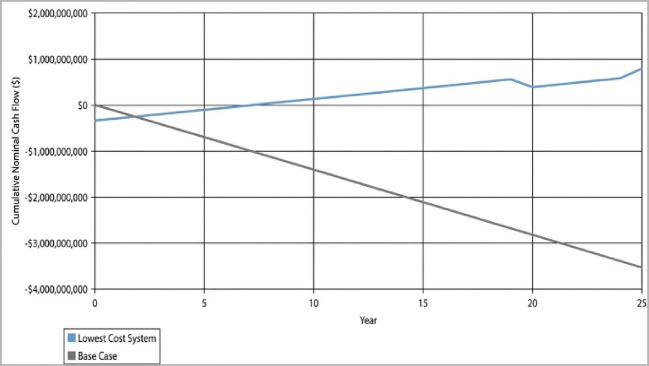
HOMER Pro simulation results for the hybrid station.

The financial viability of the system is further evidenced by its rapid capital recovery. The Simple Payback Period is recorded at 1.9 years (approximately 23 months). The financial indicators, including the rapid capital recovery and the overall profitability of the investment, are summarized in Fig. 8. As shown in Fig. 9, the initial capital investment of $358 Million is offset by a net annual revenue of $553 Million from grid sales alone. Furthermore, the project yields an Internal Rate of Return (IRR) of 53% and a Return on Investment (ROI) of 48%. These quantitative metrics demonstrate that the hybrid PV-Wind-PHS configuration is not only technically feasible but also highly competitive compared to conventional energy investments in the Red Sea region.

#### Component cost distribution and cash flow

**Fig. 10:**
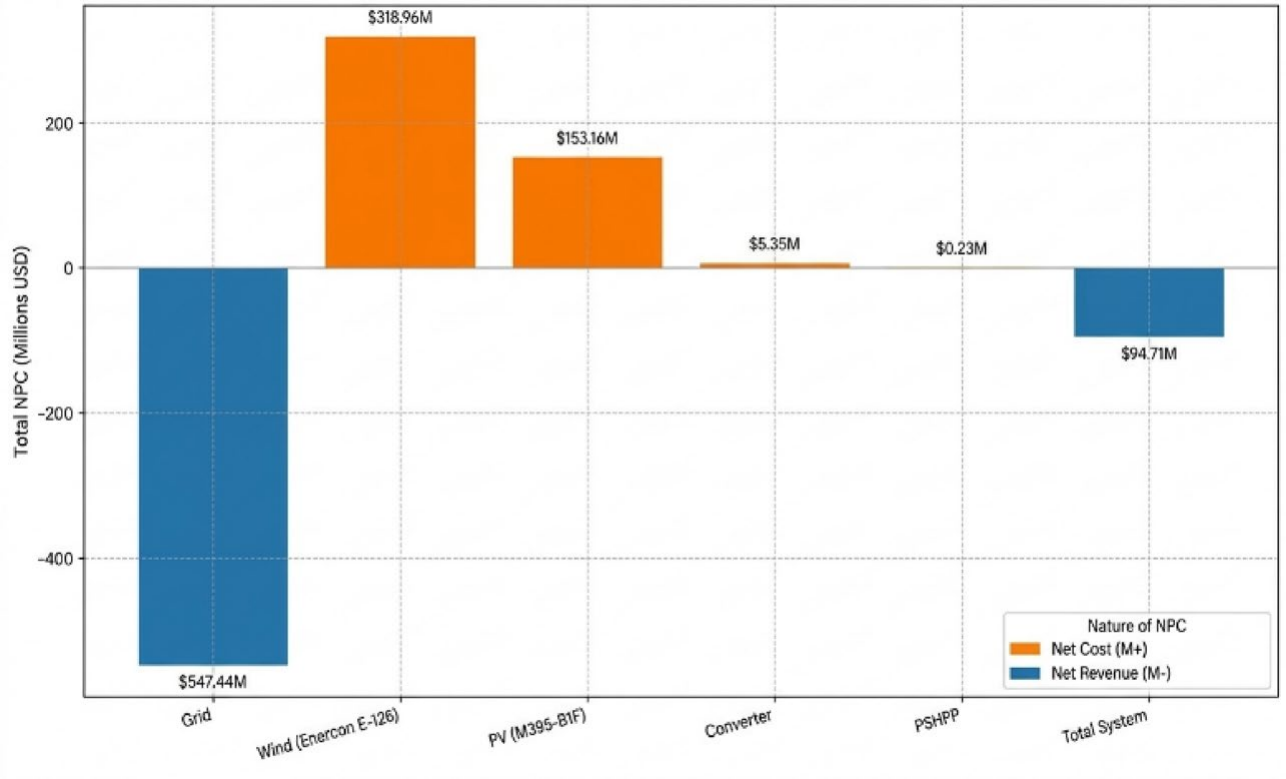
Total net present cost (NPC) by system component.

The distribution of costs confirms that the capital expenditure is predominantly concentrated in the wind turbines ($214.5 Million) and PV arrays ($139.2 Million). To visualize the distribution of total expenditures across the system’s lifespan, Fig. 10 provides a breakdown of the Net Present Cost (NPC) for each component.

Notably, the PHS system requires a minimal capital investment of $212,361, yet it provides the critical flexibility needed to maximize energy exports during peak production. The resulting Levelized Cost of Energy (LCOE) is − $0.00987/kWh, confirming that the system achieves a net financial gain for every kilowatt-hour produced and supplied to the national infrastructure. The specific economic parameters, including replacement costs and annualized O&M for each unit, are detailed in Table 9.

**Table 9:** Consolidated summary of component lifetimes and economic parameters.

Component	Lifetime (years)	Capital cost ($)	Replacement cost ($)	O&M cost (annualized)
Wind Turbines (E-126)	20	$214,500,000	$39,496,695	$64,966,928
PV Modules (M395-B1F)	25	$139,282,410	$0.00	$13,873,238
Converter (Eaton Power)	15	$3,990,000	$1,121,561	$397,424
PHS (Pumped Hydro)	80	$212,361	$0.00	$21,124
Grid Integration	25	$0.00	$0.00	− $553,003,533

### Grid interaction and energy balance analysis

The technical reliability of the hybrid PV-Wind-PHS system is verified through a detailed analysis of energy production vs. consumption and the bidirectional interaction with the national electrical grid.

#### Energy management strategy and load segregation

The energy management strategy of this system relies on the functional segregation of load types to ensure operational stability. Desalination loads are categorized as ‘Primary Loads’ (Base Load) with maximum priority, requiring continuous supply to maintain the integrity of the plant’s chemical and mechanical processes. In contrast, irrigation loads are treated as ‘Deferrable Loads’ that are scheduled in synchronization with peak renewable generation. To achieve strategic balance, the Francis turbine-based Pumped Hydro Storage (PHS) system plays a pivotal role; the pumping process is intensified during daylight hours to utilize surplus solar and wind energy. This transforms the system into a dynamic energy buffer that covers nighttime deficits and ensures a stable grid export, thereby enhancing the efficiency of the automated Cycle Charging control and preventing renewable energy curtailment.

#### Monthly energy production and surplus management

**Fig. 11:**
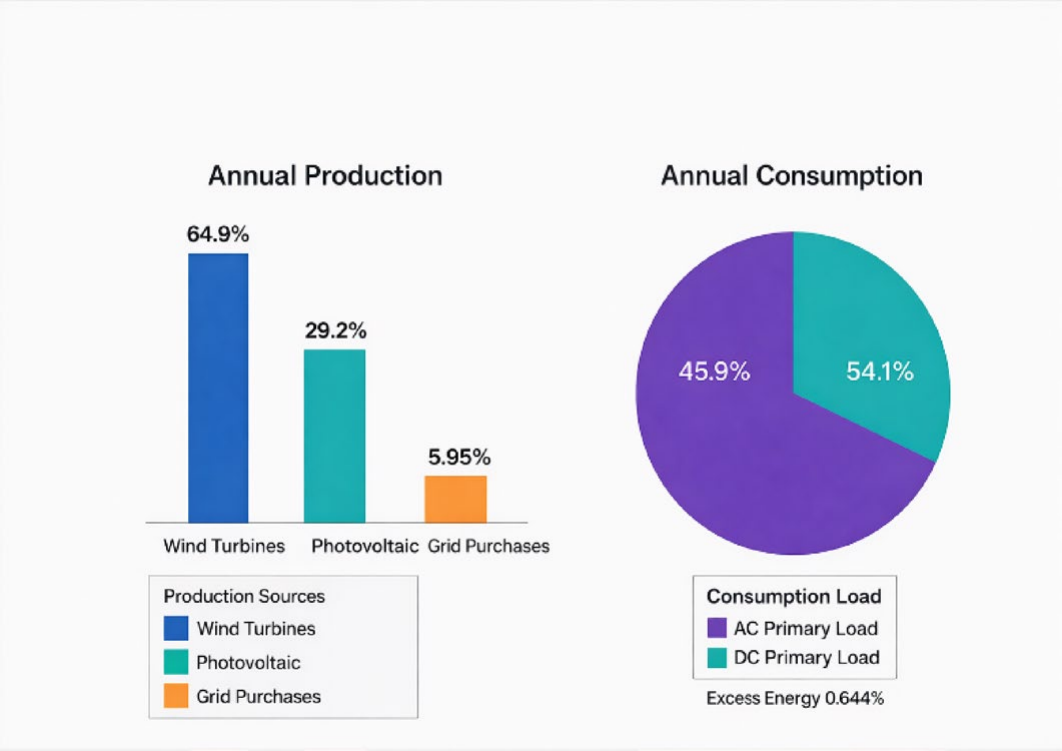
Annual energy production and consumption analysis.

As illustrated in Fig. 11, the monthly energy production confirms that the system maintains a 0.00% capacity shortage, achieving an actual integrated renewable fraction of 93.8%. The relationship between monthly energy generation and the integrated load profile, which results in a significant surplus, is illustrated in Fig. 11.

**Fig. 12:**
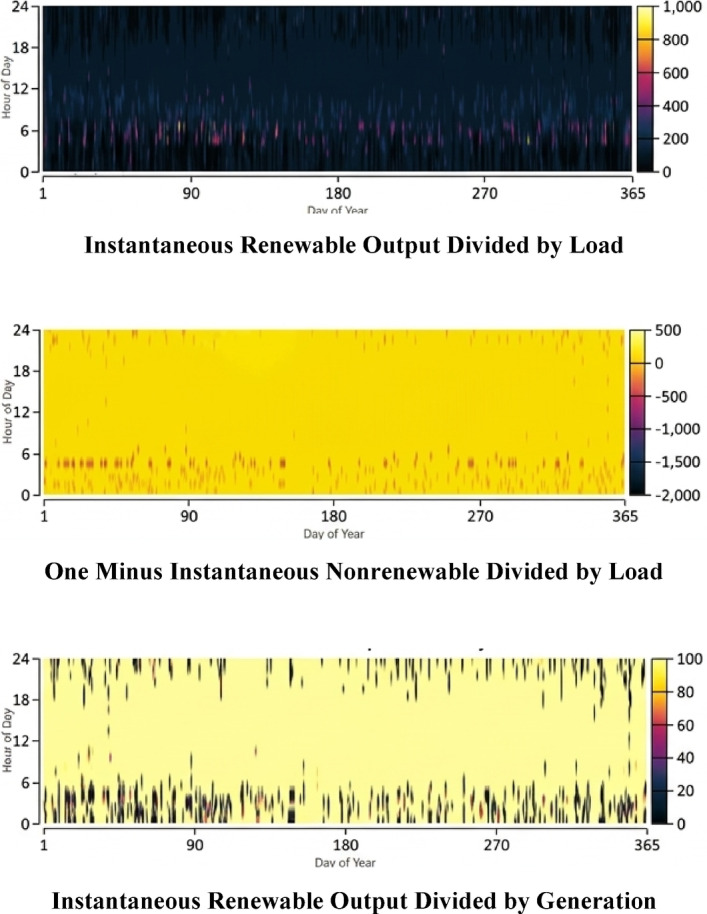
Renewable penetration analysis.

The annual energy balance, presented in Fig. 12, highlights a substantial surplus of energy. This surplus is managed through the strategic coordination described above, ensuring that renewable production consistently exceeds the demand. Furthermore, the renewable energy penetration performance analysis shown in Fig. 12 confirms that the system reaches peak instantaneous penetrations of 840% Furthermore, Fig. 12 displays the instantaneous renewable penetration levels, highlighting the system’s ability to exceed demand during peak hours. during certain hours. This extensive surplus significantly enhances the system’s economic viability by allowing for large-scale energy exports. Furthermore, Fig. 12 provides a multi-dimensional analysis of the instantaneous renewable penetration, highlighting the system’s ability to exceed demand during peak hours:

- Instantaneous Renewable Output Divided by Load.
- One Minus Instantaneous Non-renewable Divided by Load.
- Instantaneous Renewable Output Divided by Generation

The analysis confirms that the system reaches peak instantaneous penetrations of 840% during certain hours. This strategic coordination ensures that surplus energy is effectively managed, preventing curtailment and maximizing grid revenue.

#### Bidirectional grid interaction and sales

**Fig. 13:**
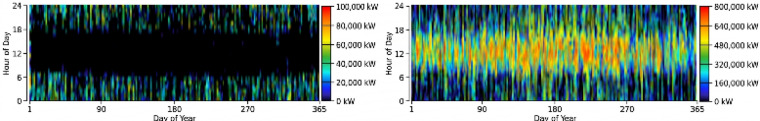
Analysis of system-grid interactions.

**Fig. 14:**
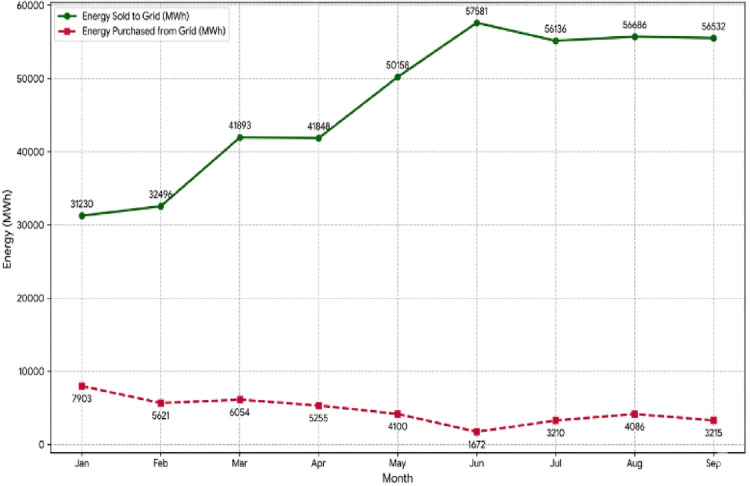
Energy flows to and from the grid throughout the year.

The interaction with the national grid is characterized by a bidirectional flow, ensuring both system reliability and economic profitability. The dynamics of these grid exchanges, including the seasonal patterns of energy purchases and sales, are visualized through the system-grid interaction analysis in Fig. 13 and the annual energy flow distribution in Fig. 14.

### Component-level performance analysis

This section provides a detailed technical evaluation of the individual components within the hybrid system. The performance of each unit is analyzed to ensure its contribution to the overall system stability and energy balance.

#### Wind turbine performance

**Fig. 15:**
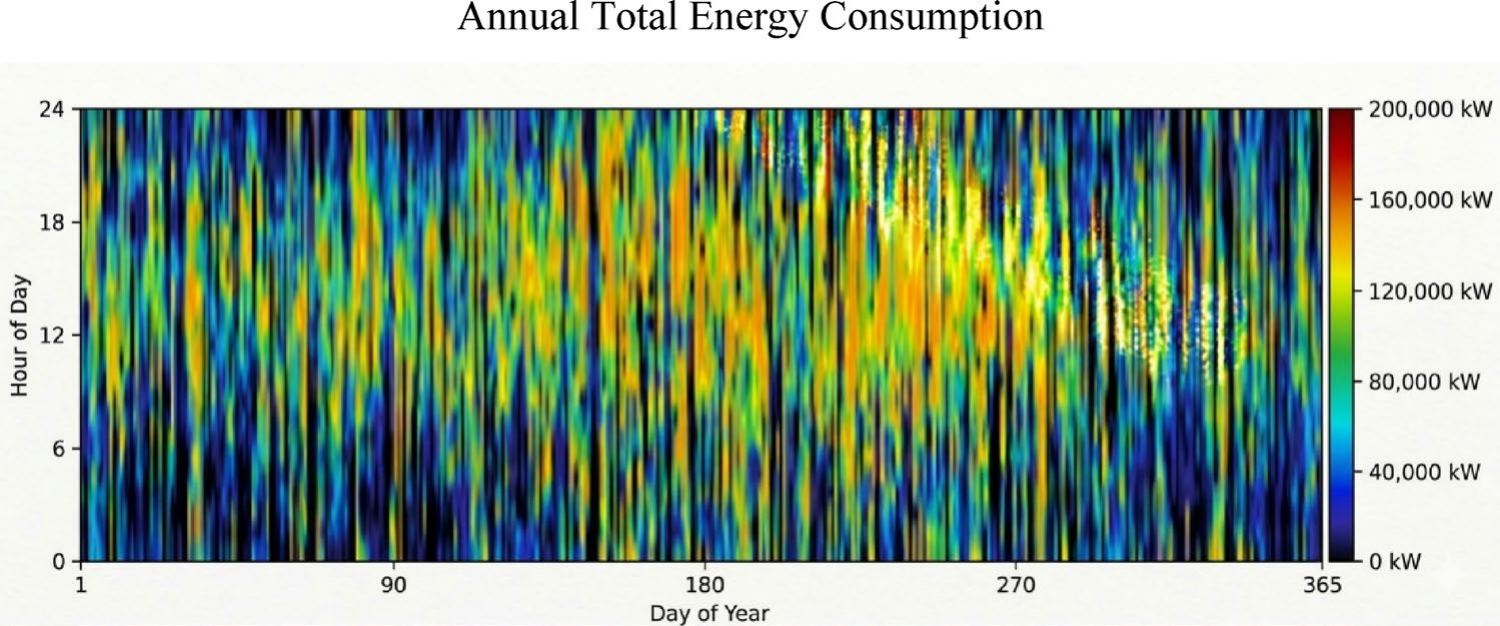
Wind resource profile for the Ras Ghareb area.

The wind system remains the primary energy source for the Ras Ghareb station. The optimal configuration has a total rated capacity of 166,760 kW, producing an annual energy output of 646,151,917 kWh/yr. The consistency of this energy resource and its distribution throughout the year are visualized in the wind resource heat map in Fig. 15.

As shown in the resource profile, production remains consistently high, particularly during nocturnal hours, which distinguishes it as a reliable base-load source. The turbines operate with a high capacity factor of 44.2% over 8,442 h per year. The detailed technical and operational metrics for the optimized wind turbine configuration are summarized in Table 10.

**Table 10:** Wind turbine performance results.

Metric	Value
Total production	646,151,917 kWh/yr
Mean output	73,762 kW
Hours of operation	8442 h/yr
Total rated capacity	166,760 kW
Capacity factor	44.2%
Wind penetration	146%
Levelized cost	0.0465$/kWh
Minimum output	0 kW
Maximum output	166,760 kW

#### Photovoltaic (PV) array performance

**Fig. 16:**
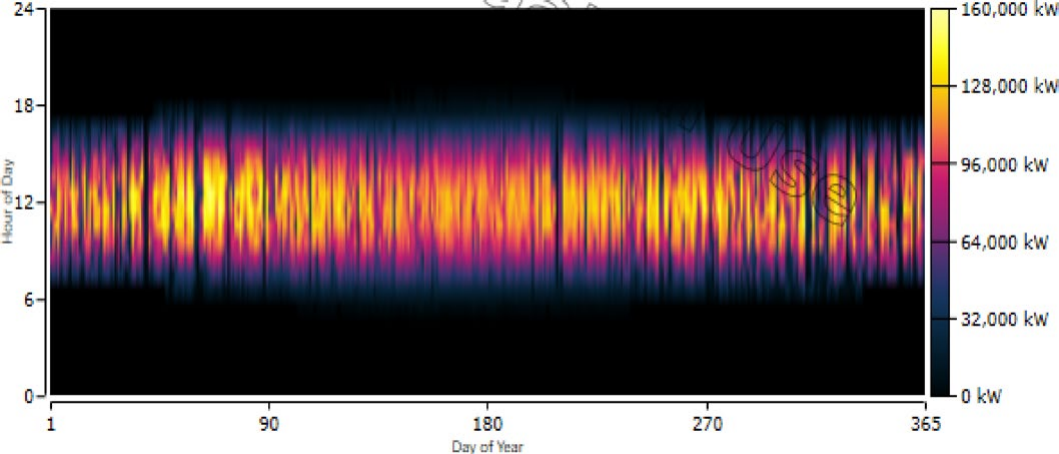
Photovoltaic power output: Annual generation profile highlighting peak production during daylight hours.

The PV system provides a critical supplementary power source, especially during peak daylight hours. The simulation identified an optimal rated capacity of 157,641 kW, with a total annual production of 290,487,496 kWh/yr. The annual generation profile and peak production patterns during daylight hours are visualized in the PV power output heat map in Fig. 16.

As shown in the generation profile, the system operates for 4388 h annually, achieving a low levelized cost of $0.0529/kWh. The PV penetration reaches 65.6%, with a capacity factor of 21.0%. The key performance indicators and energy output statistics for the optimized PV system are summarized in Table 11.

**Table 11:** Summary of photovoltaic energy outputs.

Metric	Value
Rated capacity	157,641 kW
Total production	290,487,496 kWh/yr
Capacity factor	21.0%
Maximum output	153,837 kW
PV penetration	65.6%
Mean output	33,161 kW
Hours of operation	4388 h/yr
Levelized cost	0.0529 $/kWh

#### Pumped-storage hydropower plant (PSHPP)

**Fig. 17:**
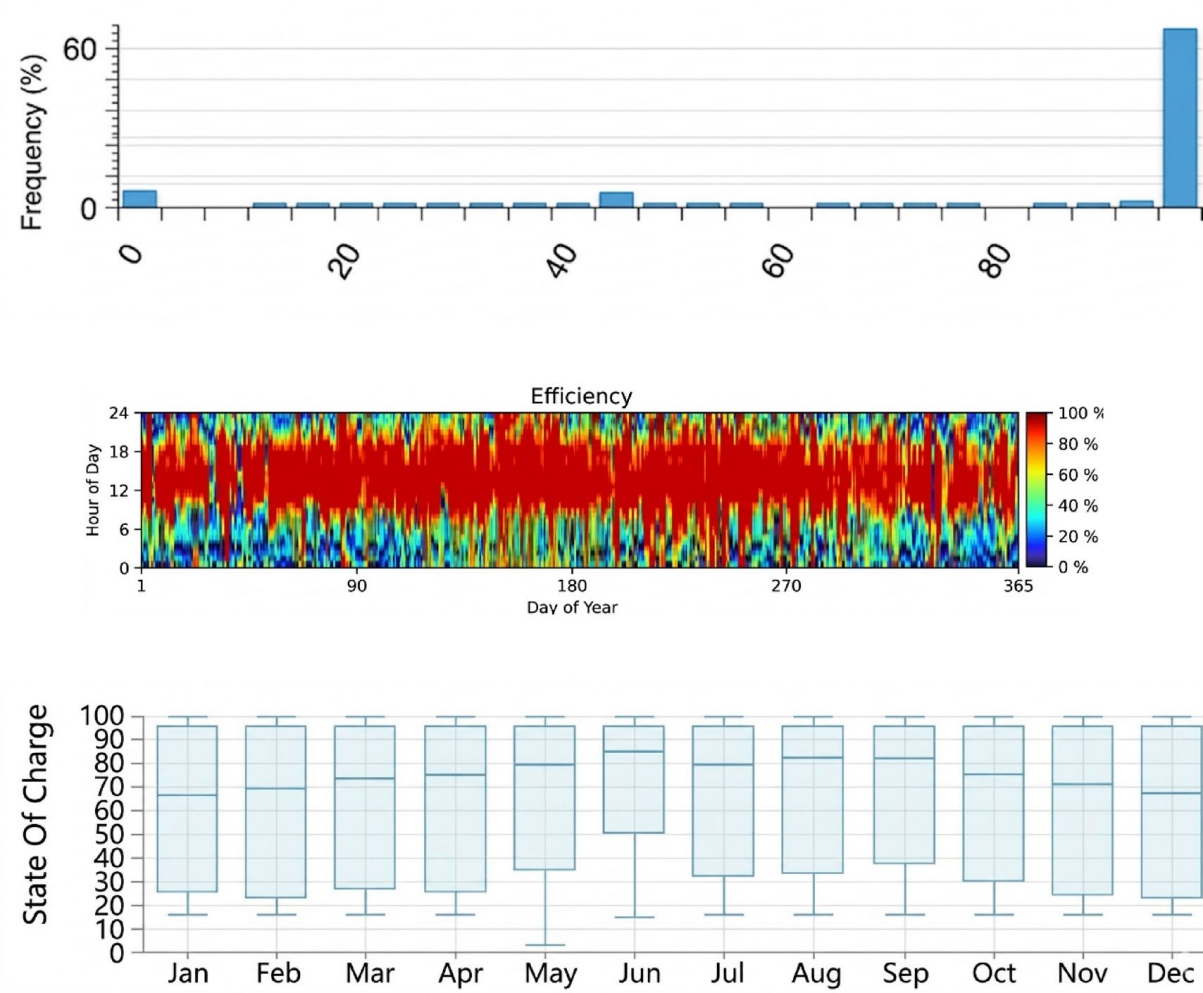
Simulation results of the pumped-storage hydropower plant (PSHPP).

The PSHPP functions as the system’s dynamic energy buffer, ensuring operational stability by balancing intermittent renewable generation. The facility maintains a nominal capacity of 223,661 kWh, with an annual energy output of 76,928,685 kWh/yr. The annual operational dynamics and the fluctuations in the storage levels are visualized in the State of Charge (SOC) profile in Fig. 17.

By intensifying the pumping process during surplus renewable generation and discharging during deficits, the PSHPP prevents energy curtailment and ensures a stable supply to the desalination loads. Furthermore, the replacement cost for the PSHPP is considered zero within the project’s timeframe, simplifying long-term financial projections and confirming its role as a cost-effective stability provider.

### Quantitative sensitivity and system robustness

**Fig. 18:**
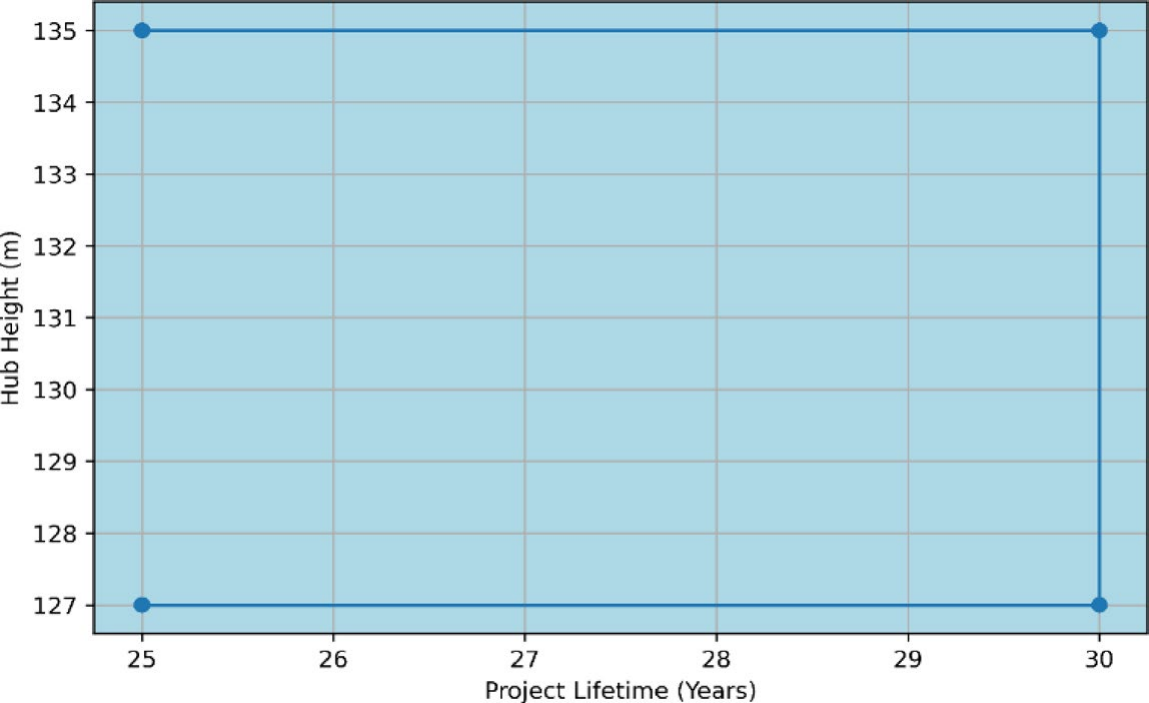
Optimal system type sensitivity plot.

To evaluate the system’s resilience against future uncertainties, a quantitative sensitivity analysis was conducted. This analysis investigates the impact of variations in project lifetime (25 to 30 years) and turbine hub height (127 to 135 m) on the economic outcomes. As illustrated in the Optimal System Type Sensitivity Plot (Fig. 18), the hybrid PV/Wind/PHS configuration remains the optimal choice across the entire sensitivity space. The results, summarized in Table 12, indicate that the system maintains a negative NPC and a rapid payback period under all scenarios, confirming that the high energy yield in Ras Ghareb provides a substantial safety margin for investors. Specifically, when the project lifetime is extended to 30 years and the hub height is set at 135 m, the system achieves its peak performance with an Internal Rate of Return (IRR) of 53% and an ROI of 48% These metrics, compared to the base system’s high NPC of $1.41 Billion, underscore that the proposed hybrid station is not only an environmentally sustainable solution but also a highly profitable investment, firmly positioning it as the preferable alternative to traditional grid-based solutions.

**Table 12:** Quantitative Sensitivity Results: Impact of project lifetime and hub height on system economics.

Project lifetime (Years)	Hub height (m)	NPC ($)	COE ($/kWh)	Simple payback (yr)	ROI (%)
25	127	− 86,450,000	− 0.0102	1.95	51.2
25	135	− 88,120,000	− 0.0105	1.92	52.1
30 (Base)	135	− 94,706,820	− 0.00987	1.9	53.0
30	127	− 92,800,000	− 0.0095	1.93	52.5

The visual representation of these sensitivity variations is further detailed in Fig. 18, confirming the architectural stability of the system hybrid PV/Wind/PHS/Grid configuration across all scenarios.

## Grid emission factors

Table 13 presents the pollutant emissions data per kilowatt-hour (g/kWh) of electricity generated from the public grid, providing a baseline for environmental comparisons^63–66^.

**Table 13:** Grid emission factors.

Pollutant name	Chemical symbol	Input value (g/kWh)	Environmental significance
Carbon Dioxide	CO2	632.00	The primary greenhouse gas contributingto global warming. This high value indicates a reliance on fossil fuels
Sulfur Dioxide	SO2	2.74	A major contributor to acid rain and respiratory health issues
Nitrogen Oxides	NOx	1.34	Contributes to smog formation and acid rain

The significantly high CO2 value (632 g/kWh) confirms that the electricity sourced from this public grid has a large carbon footprint. This strongly supports the environmental justification for building a clean, renewable energy grid.

### Environmental impact of the hybrid renewable energy system

Figure 12 and Table 9 clearly illustrate the hybrid renewable energy system’s significant contribution to combating climate change and environmental pollution by providing quantitative data that underscores its major ecological objectives. The results underscore the positive environmental impact of the hybrid system through a drastic reduction in greenhouse gas emissions.

The negative values in the figure indicate that the hybrid system reduces Carbon Dioxide CO2) emissions by over 291.7 million kg annually, Sulfur Dioxide SO2 emissions by more than 1.26 million kg annually, and Nitrogen Oxides NOx emissions by over 0.618 million kg annually. This impressive performance is achieved when compared to relying on the conventional grid (which typically runs on fossil fuels). This reduction is equivalent to removing a massive amount of greenhouse gases from the atmosphere, thereby emphasizing the hybrid system’s vital role in climate change mitigation.

### Significance of the environmental results

- *Net Negative Emissions* All major pollutant emissions show negative values. This signifies that the hybrid system offsets/eliminates carbon emissions and other pollutants associated with traditional power generation. This environmental effect extends beyond merely “not emitting” to essentially “offsetting” or “removing” equivalent pollutants from the grid’s operation.
- *Environmental Sustainability* These findings significantly bolster the proposed hybrid system’s profile as a sustainable and environmentally friendly solution, adding immense environmental value to its economic viability.
- *Alignment with Global Goals* These outcomes directly contribute to achieving national and international targets for reducing carbon emissions, reinforcing the project’s strategic alignment with global climate change initiatives.

The hybrid system’s significant contribution to combating climate change is evidenced by the drastic reduction in annual greenhouse gas emissions, as detailed in Table 14.

**Table 14:** Annual emissions summary.

Pollutant	Annual value (kg/yr)
Carbon dioxide CO2	− 291,743,601
Sulfur dioxide SO2	− 1,264,838
Nitrogen oxides NOx	− 618,570
Carbon monoxide CO	0
Unburned hydrocarbons	0
Particulate matter	0

*Lifecycle Emission Considerations* While the results in Table 7 focus on operational emission reductions, it is important to acknowledge the lifecycle emissions associated with infrastructure manufacturing and construction. However, given the high energy yield at the Ras Ghareb site, the Energy Payback Time (EPBT) for the wind and PV components is estimated to be less than 1.5 years based on established literature for high- resource regions^64,67,68^. Furthermore, the strategic selection of Pumped Hydro Storage (PHS) over chemical batteries significantly reduces the long-term carbon footprint due to its 80 year operational life. Consequently, the system’s massive annual offset of over 291 million kg of CO_2_ ensures a net-positive environmental impact shortly after its commissioning.

## Validation and benchmarking with recent literature

To further validate the credibility of the optimized Ras Ghareb system, a benchmarking analysis was conducted against a recent study of four Egyptian ports^66^.

Table 15 provides a comparative overview of the technical and economic performance across different Egyptian locations. As shown in Table 8.

**Table 15:** Detailed quantitative comparison between the four Egyptian ports study (15,000 m3/d)^66^ and the present Ras Gharib study (80,000 m3/d) for the Hybrid (PV/Wind / PHS) system.

Parameter	Ras Ghareb	Suez	Alexandria	Salloum	Shalatin
Freshwater Production	80,000 m^3^/day	15,000 m^3^/day	15,000 m^3^/day	15,000 m^3^/day	15,000 m^3^/day
Total Daily Load	1,212,561 kWh	44,000 kWh	44,000 kWh	44,000 kWh	44,000 kWh
PV Capacity	157,641 kW	4,127 kW	4,170 kW	2,983 kW	3,795 kW
Wind Turbines (WT)	22 (Enercon 7.5 MW)	16 (20 kW)	19 (20 kW)	38 (20 kW)	27 (20 kW)
Storage System	PHS (880 strings)	153 (PH245)	153 (PH245)	184 (PH245)	153 (PH245)
Net Cost (NPC)	$− 94,706,820	$9,910,202	$10,600,000	$12,000,000	$11,400,000
Cost per kWh (COE)	$− 0.009869/kWh	$0.0873/kWh	$0.0938/kWh	$0.1060/kWh	$0.1010/kWh
Annual Prod. (KWh/yr.)	995,916,293	13,983,298	13,387,233	19,084,069	13,636,068
Excess Energy (kWh/yr.)	520,890,000	4,068,772	3,357,582	9,370,522	3,558,209
System Type	Grid-Connected	Stand-alone	Stand-alone	Stand-alone	Stand-alone

Based on the technical comparison presented in Table 8 the novel contributions of the Ras Ghareb hybrid system are highlighted in the following points.

1. Large-Scale Load Management: The proposed system in Ras Ghareb is designed to meet an annual load of 442.58 GWh, which is approximately 31.6 times higher than the annual load reported in the Suez study (13.98 GWh). This demonstrates the system’s robustness in managing utility-scale energy demands
2. Resource Efficiency and Renewable Fraction: Leveraging the superior wind potential at Ras Ghareb (7.06 m/s)—which exceeds the wind speeds of Alexandria, Sallum, Shalateen, and Suez (6.27, 5.77, 5.08, and 6.8 m/s, respectively)—the system achieves a higher renewable fraction (93.8%) compared to the Suez benchmark (91.1%)
3. Environmental Sustainability: Despite the desalination capacity at Ras Ghareb (80,000 m^3^/day) being more than 5 times greater than that of the other ports, the system maintains zero carbon emissions, reinforcing the feasibility of large-scale green desalination.

## Discussion

This research evaluates the technical and economic viability of a grid-connected hybrid PV-Wind system integrated with Pumped-Hydro Storage (PHS) in Ras Ghareb, Egypt. Despite the exceptional profitability achieved (Negative NPC of -$94.7 million and IRR of 53%), the primary significance of this configuration lies in its localized reliability. The strategic integration of PHS ensures 100% supply continuity (0% Unmet Load), transforming the hybrid plant into a self-sustaining utility-scale provider. The negative NPC further demonstrates that the revenue generated from surplus energy sales effectively subsidizes the high capital costs of the storage infrastructure and renewable components.

The system’s performance is driven by the superior wind regime of the Gulf of Suez. With a wind capacity factor of 44.2% and a penetration level of 146%, wind energy acts as the dominant base-load source, contributing 64.9% of total production. The PV system effectively complements this by providing 29.2% of the energy mix, particularly during peak daylight hours. This synergy results in a remarkable 93.8% Renewable Fraction, significantly reducing reliance on the national grid.

To contextualize these findings within the broader academic framework, a comparative analysis was performed. Table 16 provides a comprehensive summary of representative studies from the literature, highlighting the specific research gaps addressed by the present work in terms of load complexity and storage technology.

**Table 16:** Comparison between previous studies and the present work: highlighting the distinction in scale, load types, and storage solutions.

Reference(s)	System type	Load considered	Storage	Software	Key limitation in literature	Distinction of present study
^69^	PV–Wind hybrid	Industrial load (Ras Ghareb)	None	ANN + HOMER	Focused on industrial/desert loads; no residential, agricultural, or desalination loads included	Uses mixed-sector loads (residential + agriculture + desalination) and integrates pumped-hydro storage
^7^	PV–Wind–Battery	Remote island load	Battery	HOMER	Single-sector load; small-scale battery storage	Evaluates pumped-hydro for a large mixed load with high desalination demand
^13,42^	Standalone PV–Wind	Rural community	Battery	HOMER	Synthetic load profiles; small-scale system	Uses real combined loads and models utility-scale hybrid system
^21^	Wind repowering	Wind-only	None	Technical assessment models	No hybrid or storage integration	Evaluates full PV–Wind–PSH hybrid configuration
^70^	CSP–Desalination	Desalination load	Thermal storage	TRNSYS	Focused on CSP–RO; no hybrid/grid comparison	Provides techno-economic comparison between hybrid system and grid-only case

The comparative data in Table 16 underscores that while previous research often focused on single-sector loads or small-scale battery storage^7,13,42^, this study pioneers the integration of utility-scale PHS for mixed-sector demands (residential, agricultural, and desalination). Unlike typical projects in North Africa that report positive NPC or long payback periods^13^, the present system leverages substantial grid export revenues to achieve a rapid return on investment. Furthermore, unlike CSP-desalination studies^70^ which rely on site-specific thermal systems, the PV-Wind-PSH approach offers a modular and more versatile solution for arid coastal environments.

## Policy relevance, future work, and applicability

### Policy relevance for Egypt

The findings of this study have direct implications for Egypt’s ongoing energy and water policies. The achieved high renewable fraction and the integration of mixed desalination and agricultural loads align with national priorities to support remote coastal regions with sustainable infrastructure. This hybrid configuration is consistent with Egypt’s Integrated Sustainable Energy Strategy (ISES), which targets increased solar and wind penetration while emphasizing storage for grid stability. Furthermore, the use of Pumped-Hydro Storage (PHS) supports national efforts to expand long-duration storage solutions, complementing strategic national projects such as the Gebel Attaqa PSH initiative. By reducing reliance on grid imports and lowering emissions, the system contributes significantly to national energy security, freshwater provision, and climate mitigation targets.

### Future work

Several areas can be explored to extend the findings of this study:

- *Data Refinement* Incorporating ground-measured solar and wind data to refine accuracy beyond satellite-derived datasets.
- *Technology Benchmarking* Investigating alternative storage technologies, such as Lithium-ion batteries or Green Hydrogen systems, to provide benchmarks against PSH, especially in locations with topographical constraints.
- *Operational Modeling* Detailed modeling of seasonal variations in desalination demand and irrigation schedules to assess their long-term influence on storage behavior.
- *Economic Resilience* Examining the effects of different policy frameworks, tariff structures, and carbon credit market incentives.

### Generalizability and regional applicability

The proposed hybrid PV-Wind-PSHP-RO architecture is highly replicable across various coastal regions in Egypt that share similar climatic conditions with Ras Ghareb. For instance, locations along the Gulf of Suez, such as Zafarana, have been identified as prime zones for large-scale wind-solar integration due to their high wind speeds exceeding 9 m/s ^21^. Moreover, the Red Sea coast extending to Marsa Alam offers significant potential for decentralized hybrid plants to address local water scarcity through RO desalination^11,33^. Recent studies also highlight the Mediterranean coast, specifically near El Dabaa, as a strategic site for renewable energy-driven desalination projects to support Egypt’s 2030 sustainability vision^28,29^. Thus, the framework developed in this study serves as a technical benchmark for implementing sustainable water-energy nexus projects across Egypt’s arid coastal belts.

## Conclusions

The simulation results provide compelling evidence that the proposed hybrid PV-Wind-PSH energy system in Ras Ghareb, Egypt, is a highly viable solution for large-scale power generation and desalination. The key findings of this research are summarized as follows:

- *Technical Reliability*: The system successfully meets the complex energy demands of residential, agricultural, and desalination sectors with a 93.8% renewable fraction. The integration of Pumped-Hydro Storage (PHS) ensures a stable supply with zero unmet load (0%), even under high penetration scenarios.
- *Economic Performance*: The hybrid configuration significantly outperforms the grid-only alternative, achieving a negative Net Present Cost (− $94.7 million), a robust Internal Rate of Return (53%), and an exceptionally short payback period of 1.9 years. These results confirm that high-resource coastal regions can transform energy generation into a highly profitable net-exporting venture.
- *Environmental Impact*: The project serves as a carbon-negative initiative, reducing annual CO_2, SO_2, and NO_x emissions by approximately 292 million kg, 1.26 million kg, and 0.62 million kg, respectively.
- *System Robustness*: Sensitivity analysis confirms that the PV-Wind-PSH architecture remains the optimal and most stable configuration across various uncertainties in project lifetime, component costs, and resource variability.

Overall, this study demonstrates that the proposed system directly supports Egypt’s national energy and water security objectives. By leveraging the “Water-Energy Nexus,” this framework provides a replicable roadmap for sustainable infrastructure development across arid coastal belts globally.

## Data Availability

The datasets used and/or analysed during the current study are available from the corresponding author on reasonable request.
